# Can Mitogenomes of the Northern Wheatear (*Oenanthe oenanthe*) Reconstruct Its Phylogeography and Reveal the Origin of Migrant Birds?

**DOI:** 10.1038/s41598-020-66287-0

**Published:** 2020-06-09

**Authors:** Erjia Wang, Dezhi Zhang, Markus Santhosh Braun, Agnes Hotz-Wagenblatt, Tomas Pärt, Debora Arlt, Heiko Schmaljohann, Franz Bairlein, Fumin Lei, Michael Wink

**Affiliations:** 1 Institute of Pharmacy and Molecular Biotechnology, Heidelberg University, Heidelberg, Germany; 2 Key laboratory of Zoological Systematics and Evolution, Institute of Zoology, Chinese Academy of Sciences, Beijing, China; 3 College of Life Sciences, UniversityMerops apiaster. J. Divers of Chinese Academy of Sciences, Beijing, China; 4 Omics IT and Data Management Core Facility, German Cancer Research Center, Heidelberg University, Heidelberg, Germany; 5 Department of Ecology, Swedish University of Agricultural Science, Uppsala, Sweden; 6 Institute of Avian Research “Vogelwarte Helgoland”, Wilhelmshaven, Germany; 7 Institute for Biology und Environmental Sciences (IBU), Carl von Ossietzky University of Oldenburg, Oldenburg, Germany; 8 Center for Excellence in Animal Evolution and Genetics, Chinese Academy of Sciences, Kunming, China

**Keywords:** Ecology, Evolution, Genetics, Molecular biology, Ecology

## Abstract

The Northern Wheatear (*Oenanthe oenanthe*, including the nominate and the two subspecies *O. o. leucorhoa* and *O. o. libanotica)* and the Seebohm’s Wheatear (*Oenanthe seebohmi*) are today regarded as two distinct species. Before, all four taxa were regarded as four subspecies of the Northern Wheatear. Their classification has exclusively been based on ecological and morphological traits, while their molecular characterization is still missing. With this study, we used next-generation sequencing to assemble 117 complete mitochondrial genomes covering *O. o. oenanthe*, *O. o. leucorhoa* and *O. seebohmi*. We compared the resolution power of each individual mitochondrial marker and concatenated marker sets to reconstruct the phylogeny and estimate speciation times of three taxa. Moreover, we tried to identify the origin of migratory wheatears caught on Helgoland (Germany) and on Crete (Greece). Mitogenome analysis revealed two different ancient lineages that separated around 400,000 years ago. Both lineages consisted of a mix of subspecies and species. The phylogenetic trees, as well as haplotype networks are incongruent with the present morphology-based classification. Mitogenome could not distinguish these presumed species. The genetic panmixia among present populations and taxa might be the consequence of mitochondrial introgression between ancient wheatear populations.

## Introduction

Maternal inheritance in most taxa (such as birds), rare recombination, and appropriate gene content make mitochondrial DNA (mtDNA) the cornerstone of phylogeographic and taxonomic studies in birds and other organisms^1–3^. In addition, mtDNA is conservative in size and organization, but nevertheless exhibits comparatively high substitution rates, which makes it a good marker for evolutionary events during the last 20 million years^4^. Moreover, methods of mtDNA analysis are well established, and do not require high-performance computing power.

Avian mtDNA is a closed circular molecule containing 13 protein coding genes (PCGs), including ATPase subunit 6 (*ATP6*) and subunit 8 (*ATP8*), cytochrome *c* oxidase subunit 1–3 (*COX1, COX2* and *COX3*), cytochrome B (*CytB*), NADH dehydrogenase subunits 1–6 and 4 l (*ND1–6* and *ND4l*); 2 ribosomal RNAs (rRNAs); 22 transfer RNAs (tRNAs) and a control region (CR). For DNA barcoding, even short stretches of DNA (e.g. <650 base pairs (bp) of cytochrome *c* oxidase subunits 1 (*COX1*)) can sometimes effectively delimit species. Accordingly, *COX1* has been used as a marker gene and confirmed the classification of >94% of bird species which had previously been classified based on morphology^5–10^. Nevertheless, single mitochondrial markers are often inappropriate to reliably reconstruct the phylogeography of populations below the species level. Concatenating several mitochondrial gene sequences obtained by traditional Sanger sequencing may improve the situation, but still does not always yield satisfactory results^11–13^. With the advance of fast moving high-throughput sequencing technologies, the field of phylogeography and population genetics might break the shackles of previous sequencing technologies as they are no longer restricted to a handful of molecular markers. It is now feasible to obtain sequences of complete mitogenomes of large sample sizes to infer the phylogeographic histories of avian species in a short time.

Wheatears (genus *Oenanthe*) are small passerines in the family Muscicapidae (Old World flycatchers and chats). They are specialized to open habitats and many of them inhabit savannah and desert ecosystems^14^. To date, the genus *Oenanthe sensu stricto* comprises 29 species^14^. The Northern Wheatear (*Oenanthe oenanthe*) breeds almost across the whole Holarctic. It consists of 3 subspecies, *O. o. oenanthe* (nominate), *O. o. leucorhoa* (the “Greenland Wheatear” hereafter), and *O. o. libanotica* (southern Europe and Asia). In this study, we focused on 3 taxa, *O. o. oenanthe, O. o. leucorhoa* and *O. seebohmi*. Breeding sites of *O. o. oenanthe* range from continental Europe and Great Britain to Siberia and Alaska, while *O. o. leucorhoa* breeds in Greenland, Iceland and the East of Canada^14^. During autumn and spring migration, these two subspecies gather at stopover sites in Northern and Western Europe^15–18^. The sedentary Seebohm’s Wheatear, however, is confined to the Atlas Mountains (Morocco) and had traditionally been included in the Northern Wheatear (*O. oenanthe*) as a distinctive subspecies (*O. o. seebohmi*). However, it has recently been separated and considered as a conspecific taxon, *O. seebohmi*^14^.

Wheatears exhibit a remarkable complexity and variety of color patterns, while they are rather congeneric with respect to display behavior, foraging, and morphological traits^19^. Small, but distinctive differences exist between *O. o. leucorhoa* and *O. o. oenanthe*. In the field, the 2 subspecies to a certain extent, can be distinguished by wing length and plumage coloration^15,20^. In line with the current classification as a separate species, *O. seebohmi*, based on its plumage coloration, can be readily told apart from *O. oenanthe*. *Oenanthe seebohmi* can be discriminated by its black throat, its black underwing-coverts and its larger proportion of white on the forehead when compared to other wheatear species. However, species status and particularly the delineation of subspecies within the wheatear genus, have been challenged for decades based on ecological, geographical, morphological and genetic characters^19,21–28^. In spite of the numerous investigations of the morphology and migration ecology of wheatears^18,19,22,29–31^, this controversial debate is still ongoing and can only be resolved by molecular data.

In this study, we reconstructed the complete reference mitogenome of the Northern Wheatear and Seebohm’s Wheatear. In order to obtain a comprehensive phylogenetic framework and robust population genetics, we investigated the mitogenome of 117 individuals from 7 breeding populations: Sweden (Ammarnäs, Uppsala, *O. o. oenanthe*), Norway (*O. o. oenanthe*), Germany (Norderney, *O. o. oenanthe*), Alaska (*O. o. oenanthe*), Iceland (*O. o. leucorhoa*), and Morocco (*O. seebohmi*). We compared the resolution power of single mtDNA markers, multiple mtDNA markers and complete mitochondrial genomes for inferring phylogenetic relationships. We further used mitogenomes of Northern Wheatears, which were sampled as passage migrants at two stop-over sites, Helgoland and Crete, to test whether we could infer their origins based on complete mitogenome sequences. Finally, we assessed whether the molecular data is in consensus with the recent morphological and ecological classification of the Northern Wheatear and its closely related species Seebohm’s Wheatear.

## Results and Discussion

### Mitogenome organization

**Figure 1:**
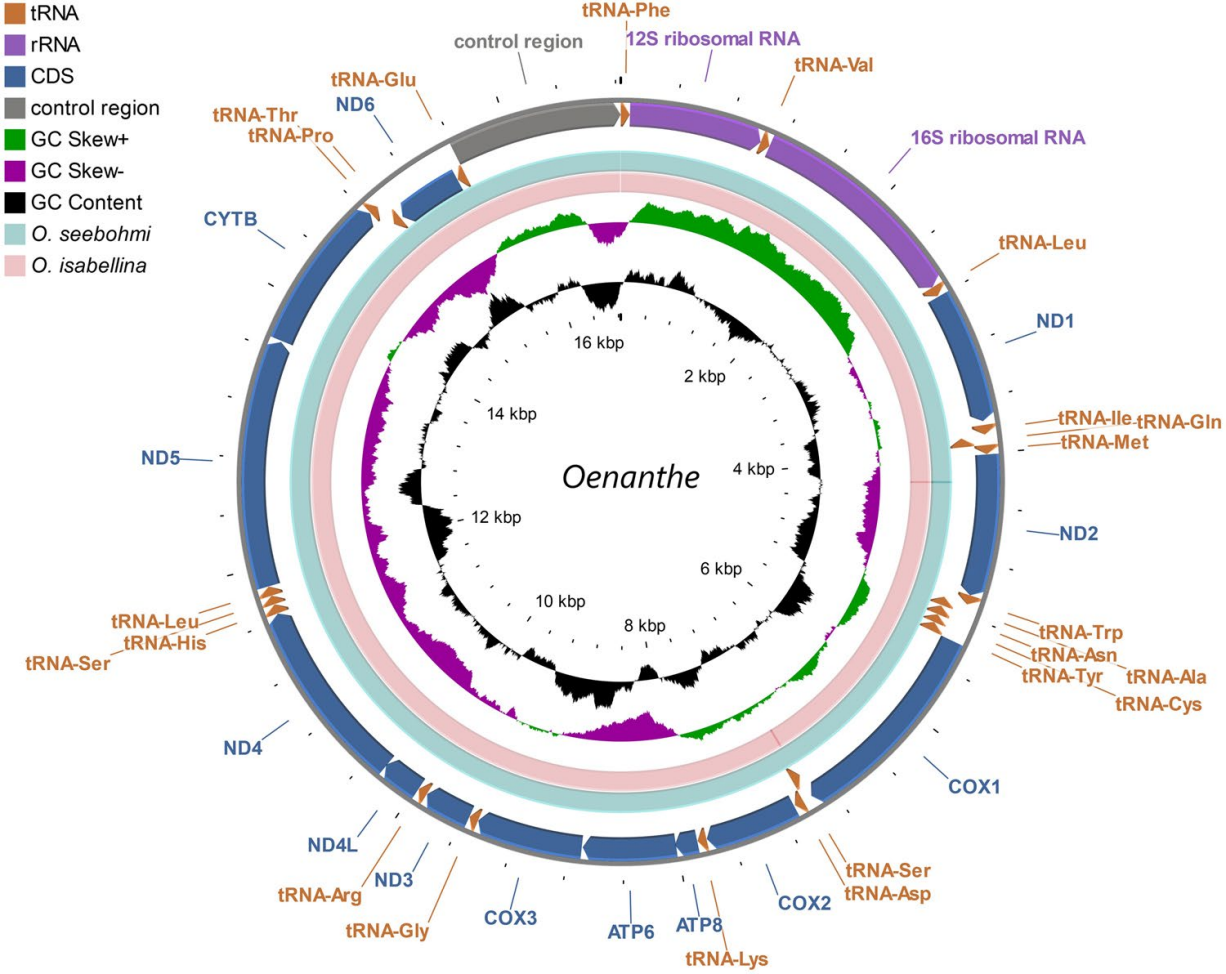
Circular map of the mitogenome of *Oenanthe oenanthe* assembled from NGS data. Features are represented by different color blocks. Arrows indicate the orientation of the gene transcription. The GC content and GC skew were calculated using a sliding window and plotted as the deviation from the average value of the entire sequence. The BLAST comparisons of *Oenanthe oenanthe* with *Oenanthe seebohmi* and *Oenanthe oenanthe* with *Oenanthe isabellina* are shown in divider rings.

Mitogenomes of 2 subspecies of the Northern Wheatear (*O. o. oenanthe* and *O. o. leucorhoa*), 1 congeneric species (*O. seebohmi*), and the outgroup *O. isabellina* were assembled *de novo* and the reads generated by massive parallel sequencing (NGS) of mtDNA were mapped. A mitogenome contraction map (Fig. 1) was built. The total length of the mitochondrial DNA sequence of *O. oenanthe* ranged from 16,824 to 16,835 bp (Table 1). Except for *ND6* and 8 tRNAs, all remaining genes [12 protein-coding genes (PCGs), 2 rRNAs and 14 tRNAs] were located on the heavy DNA strand (H-strand). The arrangement of the whole mitogenome of *O. oenanthe*, *O. seebohmi* and *O. isabellina* were identical to the typical mitogenome of birds, that was initially established in *Gallus gallus*^32^. No gene rearrangements and duplications were found in our samples. However, when compared to the *O. oenanthe*, a few deletions were detected in the mitogenome of *O. isabellina* inside the non-coding area between *ND1* and tRNA-Ile. Another deletion was found in the coding area of tRNA-Trp (Table 1). The overall base compositions of *O. oenanthe* and *O. seebohmi* under study were also similar to the related species *O. isabellina*. The relative abundance of nucleotides was C (32.8%) > A (30.0%) > T (22.9%)> G (14.3%), and the GC skew was negative for the mitogenome.

**Table 1:** Annotation of assembled mitochondrial genome of *Oenanthe oenanthe*.

Product	Start	End	Size	Strand	Name	Anti-code	Start codon	Stop codon
tRNA-Phe	1	68	68	+	trnF	GAA		
12S ribosomal RNA	69	1048	980	+	rrnS	−	AAA	TAC
tRNA-Val	1049	1118	70	+	trnV	TAC		
16S ribosomal RNA	1119	2715	1597	+	rrnL	−	TGC	CCC
tRNA-Leu	2716	2790	75	+	trnL2	TAA		
ND1	2796	3773	978	+	nad1	−	ATG	AGA
tRNA-Ile	3794	3865	72	+	trnI	GAT		
tRNA-Gln	3871	3941	71	−	trnQ	TTG		
tRNA-Met	3941	4009	69	+	trnM	CAT		
ND2	4010	5049	1040	+	nad2	−	ATG	TA(A)
tRNA-Trp	5050	5120	71	+	trnW	TCA		
tRNA-Ala	5122	5190	69	−	trnA	TGC		
tRNA-Asn	5195	5267	73	−	trnN	GTT		
tRNA-Cys	5268	5334	67	−	trnC	GCA		
tRNA-Tyr	5334	5404	71	−	trnY	GTA		
COX1	5406	6956	1551	+	cox1	−	GTG	AGG
tRNA-Ser	6948	7022	75	−	trnS2	TGA		
tRNA-Asp	7026	7094	69	+	trnD	GTC		
COX2	7102	7785	684	+	cox2	−	ATG	TAA
tRNA-Lys	7787	7854	68	+	trnK	TTT		
ATP8	7856	8023	168	+	atp8	−	ATG	TAA
ATP6	8014	8697	684	+	atp6	−	ATG	TAA
COX3	8703	9486	784	+	cox3	−	ATG	T
tRNA-Gly	9487	9555	69	+	trnG	TCC		
ND3	9556	9906	351	+	nad3	−	ATG	TAA
tRNA-Arg	9908	9977	70	+	trnR	TCG		
ND4L	9979	10275	297	+	nad4l	−	ATG	TAA
ND4	10269	11646	1378	+	nad4	−	ATG	T
tRNA-His	11647	11717	71	+	trnH	TCG		
tRNA-Ser	11718	11784	67	+	trnS1	GCT		
tRNA-Leu	11784	11854	71	+	trnL1	TAG		
ND5	11855	13672	1818	+	nad5	−	ATG	AGA
CYTB	13681	14823	1143	+	cob	−	ATG	TAA
tRNA-Thr	14827	14895	69	+	trnT	TGT		
tRNA-Pro	14902	14971	70	−	trnP	TGG		
ND6	14989	15507	519	−	nad6	−	ATG	TAG
tRNA-Glu	15509	15580	72	−	trnE	TTC		
control region	15581	16824-16835		+	CR	−		

The total length of the 13 PCGs was 11,405 bp, representing approximately 67.8% of the entire mitogenome of 3 study taxa. The GC content was 48.1% and 47.1% for the 13 PCG and the complete mitogenome, respectively. The genes coding for individual tRNAs ranged from 67 bp to 75 bp and summed up to 1,547 bp in total length. Since tRNAs play an important role in translating mRNA into protein, they are highly conserved. Accordingly, polymorphisms among birds of the 7 different breeding sites were only detected in 5 out of 22 tRNAs (tRNA-Trp, tRNA-Asn, tRNA-Lys, tRNA-Arg and tRNA-Gly). tRNAs adopted the conventional secondary structure of four-armed cloverleaves and L-shaped tertiary structure^33^. Owing to the absence of the dihydrouridine arm (D-arm), tRNA-Ser (GCT) did not fold into a cloverleaf conformation. This pattern has been known for other species for decades and is particularly common in animal mitochondrial genomes^34^.

### Phylogeography and population genetics based on the mitogenomic data

**Figure 2:**
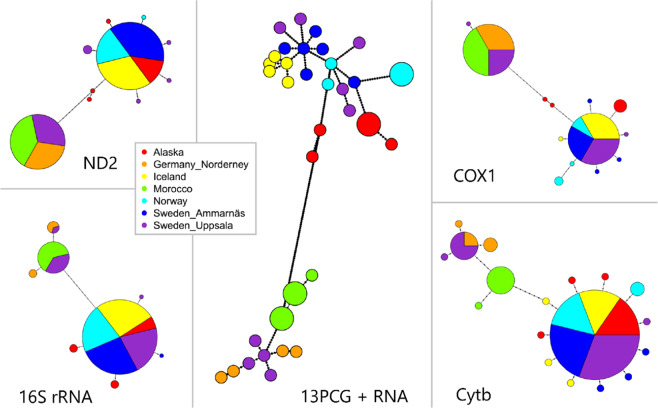
Haplotype network for 38 individuals of *Oenanthe*. Size of the circles represent the frequency of haplotypes. The breeding areas are coded by the colors. Each dot indicates one mutation step. Samples from Morocco are Seebohmi Wheatears (*Oenanthe seebohmi*); samples from Iceland are Greenland Wheatears (*O. o. leucorhoa*); samples from Alaska, Germany, Norway and Sweden are nominate wheatears (*O. o. oenanthe*). The classification of (sub)species are identified by the morphological data.

Minimum spanning network analysis of the 13 PCGs, rRNAs and tRNAs from 38 individuals from the 7 breeding populations in Central and Northern Europe (Norway, Germany_Nordeney, Sweden_Ammarnäs and Sweden_Uppsala), Iceland, Alaska and Morocco yielded 34 haplotypes with a maximum of 98 mutation steps (Fig. 2). The highest level of haplotype diversity with maximum mutation steps for single markers was found in *ND5* (Fig. S1, Fig. S2). Most of the genetic markers exhibited two haplotype groups (Figs. 2, S2). Exceptions were tRNAs and *ATP8* being either highly conservative or extraordinarily short in length (Fig. S2, Table 1). Figure 2 illustrates the minimum spanning networks based on 4 commonly used markers in phylogeography and a concatenated set containing all mitochondrial markers. When looking at the haplotype networks based on the single markers, the Norwegian *O. o. oenanthe* shared the haplotypes with the other nominate Northern Wheatears from Ammarnäs (Sweden) and Alaska, but also with the “Greenland Wheatears” (*O. o. leucorhoa*) breeding in Iceland. Hence, this first haplotype group consisted of mixed samples of *O. o. oenanthe* and *O. o. leucorhoa*. Similarly, the Moroccan *O. seebohmi* clustered with the *O. o. oenanthe* from Uppsala (Sweden) and Norderney (Germany) and constituted the second group of haplotypes. Even when using the full resolution power of all mitochondrial markers, no clear separation among the (sub)species was evident. Accordingly, a limited relationship between the geographic subdivision and haplotype classification was observed. Indeed, these star-like topologies, i.e., a few dominant haplotypes surrounded by less frequent mutations (Fig. 2, S2, Table S1), have often been reported in Eurasian birds^11–13^.

**Figure 3:**
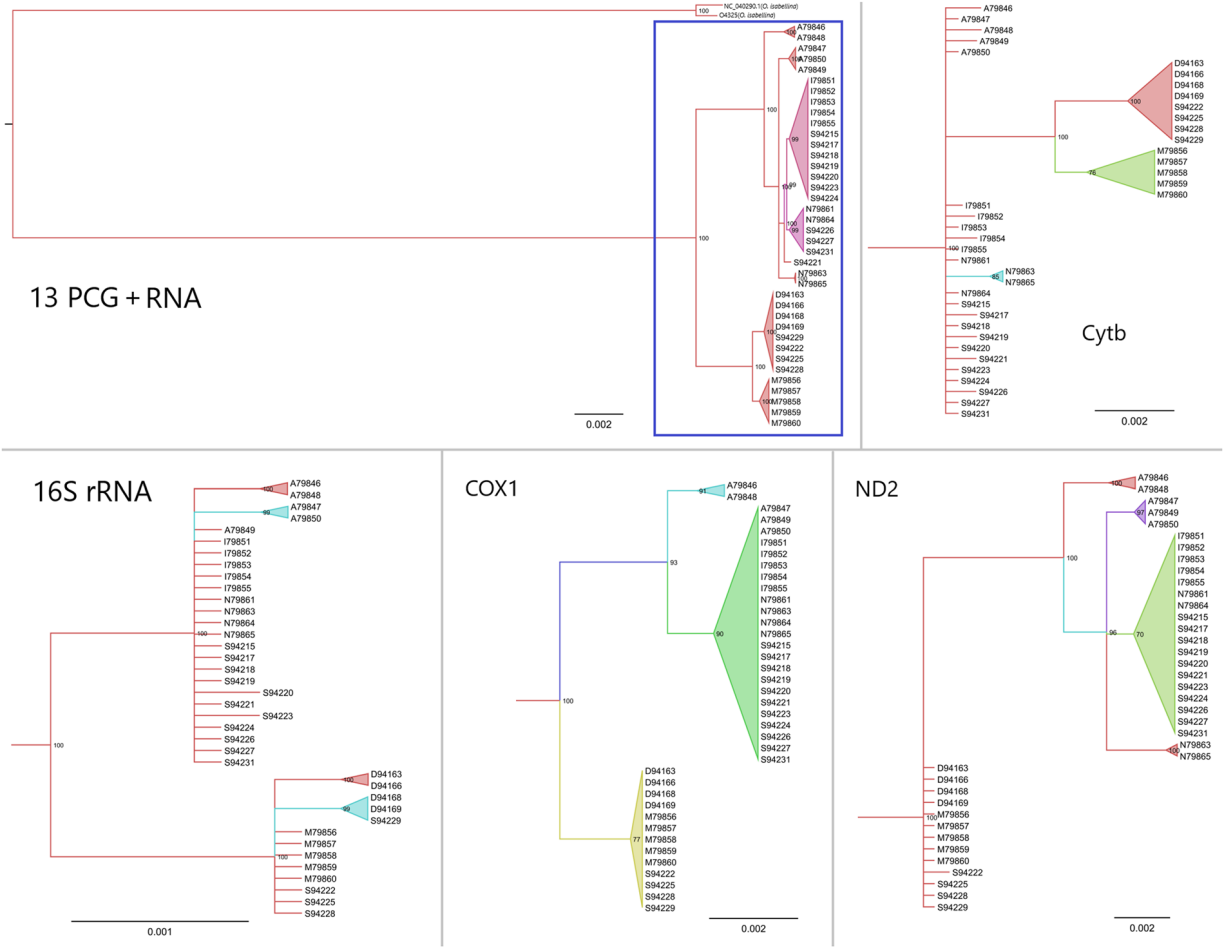
MrBayes reconstruction of the *Oenanthe oenanthe* and it’s outgroup *Oenanthe isabellina*. Numbers above nodes refer to the support values of Bayesian posterior probability. Sample name indicate the localities by the capital letter, ‘A’ refers to Alaska; ‘D’ refers to Germany; ‘I’ refers to Iceland; ‘M’ refers to Morocco; ‘N’ refers to Norway and ‘S’ refers to Sweden. Samples from Morocco are black Seebohmi Wheatears (*Oenanthe seebohmi*); samples from Iceland are Greenland Wheatears (*O. o. leucorhoa*); samples from Alaska, Germany, Norway and Sweden are nominate wheatears (*O. o. oenanthe*).

In order to calculate their evolutionary divergence, we firstly estimated the substitutions per site for all the mitochondrial markers of 38 breeding Northern Wheatears. In general, RNA genes were more conservative, resulting in low substitution rates. Among the 13 PCGs, *ND2*, the most commonly used genetic marker showed the highest substitute rate, followed by *ND5*, *ND6* and *Cytb* (Fig. S1). On the contrary, other frequently-used barcoding genes (16S rRNA and *COX1*), exhibited low levels of divergence among the Northern Wheatear populations (Fig. S1). In a next step, we compared the tree topologies of single molecular markers with the phylogenetic tree based on the concatenated set of all mitochondrial markers. We found that all the individual markers could differentiate between *O. oenanthe* and its sister species *O. isabellina*, but at the subspecies or population levels, the use of single or a few concatenated markers yielded contradictory subdivisions and shallow trees (Fig. 3, S3). Figure 3 shows inconsistent tree topologies generated from each of four frequently used markers in avian phylogeny studies (16S rRNA, *COX1*, *Cytb* and *ND2*). 16S rRNA and *COX1* had reduced substitution rates when compared to *ND2* and *Cytb*, but exhibited high congruence with the concatenated set of markers and separated the two main haplotype clusters.

For phylogenetic analysis, we followed the most common of the traditional approaches by concatenating two marker genes per analysis, namely *ND2* | *Cytb* and 16S rRNA | *COX1*. The phylogenetic tree obtained from concatenated *ND2* | *Cytb* was in agreement with the mitogenome results (Fig. S4, Fig. 3, Fig. S3). Furthermore, this combination was much more superior in terms of resolution power, when compared to the tree reconstructions based on the single markers. On the other hand, concatenated 16S rRNA | *COX1* was not superior to the individual markers 16S rRNA and *COX1*, respectively, in terms of resolution (Fig. S4, Fig. 3, Fig. S3). Since our analysis clearly indicated conflicting phylogeographies based on the use of different single or a few concatenated mitochondrial markers (Fig. 3, Fig. S3, Fig. S4), the significance of studies based on traditional approaches should be critically re-examined as they might not mirror actual evolutionary processes. Complete mitogenomes however – as shown in this study – yield potentially enhanced phylogeographic resolution at species and population levels^35–37^.

In this regard, it is important to mention that high transition-transversion ratios of avian mitochondrial DNA result in a skewed base composition^38^. For this reason, model selection taking into account codon positions greatly impacts efficiency and accuracy of mtDNA analyses^39–41^. Accordingly, the comparison of mitochondrial gene trees based on partitioned and non-partitioned sequence data, respectively, (Fig. S4) confirmed that partition strategies can increase accuracy of phylogenetic predictions as well as statistical support^42–44^.

### Can mitogenomes reveal the breeding origin of migrants captured at stop-over sites

Not only for studying phylogenetics, phylogeography and population genetics, the mitochondrial markers have also regarded as logbooks for migration studies. Different mitochondrial lineages can be distinguished by accumulating mutations over time. Based on these mutations, individuals can be assigned to those lineages and their corresponding commons ancestors^45^. The haplotype of a migrant bird might therefore theoretically predict its geographic origin. In an ideal situation, populations with limited inter-lineage gene flow, contain conservative haplotypes or closely related haplotypic groups corresponding to their geographic localities. The breeding area of migrants could then be recognized by matching to the known breeding population haplotypes^45^. One of the purposes of this study is to test this scenario. We planned to predict the origins of migrating Northern Wheatears sampled on Helgoland (Germany) and Crete (Greece) through comparing the haplotypes with those of breeding wheatears.

**Figure 4:**
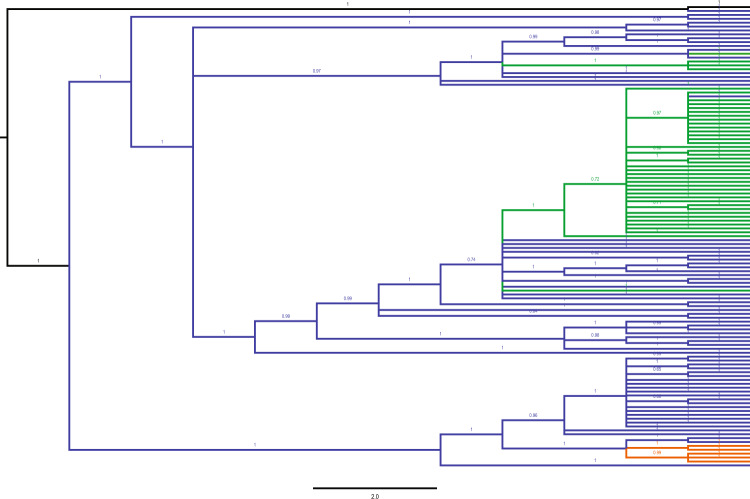
MrBayes reconstruction based on all the concatenated mitochondrial markers of 3 taxa of *Oenanthe* and its outgroup. 38 breeding samples and 79 migrant samples (caught in Helgoland and Crete) were included. Numbers above nodes refer to the support values of Bayesian posterior probability. Taxa identifications are color coded based on the field data: outgroup (black), *O. o. oenanthe* (blue), *O. o. leucorhoa* (green) and *O. seebohmi* (orange).

Given the present results, it was very unlikely that the origin of the migrant birds captured on the islands of Helgoland and Crete could be determined, since even birds from different breeding populations could not reliably be separated from each other. However, inadequate sampling^46,47^ could have contributed to the tree topology of Fig. 3. In order to minimize these effects, we included the Helgoland and Crete birds. These individuals consisted of 2 subspecies *O. o. oenanthe* and *O. o. leucorhoa*, that have been identified by morphology and whole genome sequencing (data in preparation). In addition, sequences of the Southern Northern Wheatear (*O. o. libanotica)*^21^ obtained from GenBank were included in the analyses. However, the inclusion of additional data did not considerably improve tree topology, and mitogenomic admixture among the subspecies was evident (Figs. 4, S5). This holds true for both molecular classifications based on the single mitochondrial markers and the whole mitogenomes. It was supported by high posterior probabilities implying gene flow among the 3 wheatear taxa (Fig. 3). Moreover, since the breeding birds did not form clear clusters in the haplotype network, the origin of the migrants could not be inferred using haplotype networks either (Fig. S6).

### Hypotheses to resolve the conflict between phenotype and haplotype

**Figure 5:**
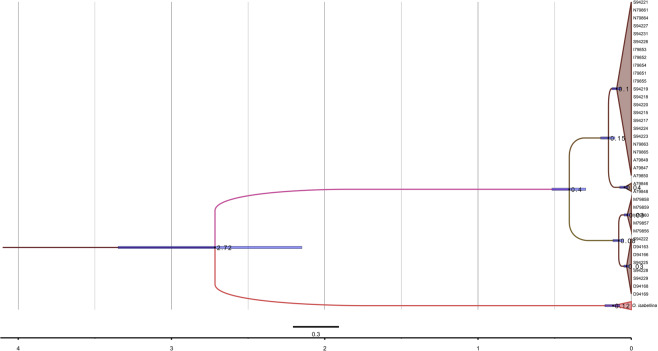
Phylogenetic relationships of *O. o. oenanthe* (blue), *O. o. leucorhoa* (green) *O. seebohmi* and *O. isabellina* based on the mitogenome. The values indicate the split time calculated by BEAST 1.8. The Bayesian posterior probabilities of all the nodes are 1. The blue bars show 95% highest posterior density (HPD) of divergence times.

In this study, we successfully detected the footprints of gene flow between ancient Northern Wheatear populations. Mitogenome data identified two clades with about 1% sequence differences, corresponding to an estimate of divergence time of about 400,000 years ago (Figs. 3–5). However, the separation of lineages did not align with the phenotypic differences. Northern Wheatears whose appearance was distinct, were genetically alike, and, vice versa. This finding stands in sharp contrast to recent taxonomy considering the Seebohm’s Wheatear as a separate species.

The explanation might be that Northern Wheatears underwent speciation reversal upon secondary contacts, causing recent introgression of mtDNA across (sub)species boundaries. Recent hybridizations between ancient populations of Morocco and Europe introduced African genetic material into the mitogenome of nominate Northern Wheatears. Due to selective sweep, these new haplotypes have become fixed in a few present populations. This hypothesis is supported by the uniparental inheritance of mtDNA, which results in a lower effective population size and correspondingly in high fixation rates of foreign mitogenomic lineages caused by introgression^48^. This is in line with multimodal mismatch distributions and non-significant neutrality tests of the 3 taxa (Fig. S7). In addition, the paleoclimatological history corroborates the assumption of speciation reversal. Past climates in the last 2 million years changed in 100,000-year cycles, with alternating warm and cold periods causing the northern hemisphere to repeatedly become inhospitable for most living species^49–52^. During the glacial periods, large parts of North America, North and West Europe were covered by a thick layer of ice. Only some ice-free Mediterranean areas served as refugia for survivors. For instance, Portugal and Spain, Italy, and the Balkans were three potential refuges during the last glaciation^53–55^. Consequently, populations of different origins regularly met and exchanged genetic material in the ice-free refugia. They could spread to different locations when ice sheets receded, and suitable new vegetation zones appeared. The hypothesis of cyclical mitochondrial introgression upon secondary contacts could explain that some ancestral mitochondrial polymorphisms are conserved in multiple lineages of recent Northern Wheatear populations and subspecies, although nuclear gene flow is limited (MS in preparation).

Inconsistencies between the mitogenome and morphology-based classifications are not a rare phenomenon. A remarkable example is yellowhammers (*Emberiza citrinella*) and pine buntings (*Emberiza leucocephalos*); these two species share similar mitochondrial haplotypes although they are morphologically divergent^56^. Similar patterns of genetic and phenotypic discordance have also been reported in the other species^57–60^. In addition to introgression, incomplete lineage sorting (ILS) can lead to non-monophyly and mito-nuclear incongruence, when the species divergence has occurred recently^61^. Indeed, it is cumbersome to distinguish these two processes because they generate similar genetic signatures^62,63^. Many recent studies have evaluated the high impact of ILS on the coalescent analysis of reconstructing the speciation process^61,64,65^. However, we do not consider the ILS as responsible for the unusual mtDNA divergence among these three (sub)species since only part of the nominate populations (*O. o. oenanthe*) shared haplotypes with the Moroccan populations (*O. seebohmi*). Therefore, it is more likely that introgressive hybridization (because of cyclic range expansions and reduction during warm and cold periods) determined the haplotype patterns of Northern Wheatears.

## Conclusion

Our study is the first one to describe the complete mitogenome of the Northern Wheatear and to reconstruct phylogeographic relationships based on mitochondrial genomes using a large sample size. Our results based on high-quality genetic data differ from the recent subspecies classification derived from morphological traits. The subspecies *O. o. oenanthe* and *O. o. leucorhoa* and even *O. seebohmi* cannot be distinguished by mtDNA. This might be the consequence of recent mitochondrial introgressive hybridizations between the taxa. High resolution markers, such as genome-wide SNPs, should be used, as they could separate these 3 (sub)species (MS in preparation). Although the mitogenome is not congruent with the morphological, ecological and nuclear classification of Northern Wheatears, it can still contribute to deciphering evolutionary processes. A big advantage of mtDNA in phylogeography is its major contribution to detecting recent historical events, which might otherwise have been overlooked if analyses are solely based on nuclear markers.

## Materials and methods

### Sample collection, DNA extraction and sequencing

In this study, we sampled 117 Northern Wheatears (*O. oenanthe*) and Seebohm’s Wheatear (*O. seebohmi*), including samples from birds breeding in Alaska (5), Iceland (5), Norway (4), Germany (4), Sweden (6 in Ammarnäs; 9 in Uppsala), Morocco (5) and samples of actively migrating birds from Greece (6 on Crete) and Germany (73 on Helgoland) (Table S2). As outgroup we used *O. isabellina*^66^. All methods carried out in accordance with the relevant guidelines and regulations. In Sweden, birds were captured and marked with permission from the Swedish Bird Ringing Centre. The sampling for Uppsala was approved by the Uppsala animal ethics committee (permit no. C117/8), and for Ammarnäs by the Umeå animal ethics committee M64–05 and M160–11). In Alaska, the sampling was under a license of the U.S. Fish and Wildlife Service (Federal Fish and Wildlife Permits: MB207892-0, MB97904A-0) and the State of Alaska Department of Fish and Game (Permits: 13–103, 14–009). In Germany, the sampling was under license of the German Federal State of Lower Saxony (33.19-42502-04-16/2349) and Schleswig-Holstein (V 244-4829/2017 (33-3/17)). The samples from Greece were provided from IPMB (Institute of Pharmacy and Molecular Biotechnology, Heidelberg University, Germany). Total genomic DNA was extracted from the blood or tissue samples according to the standard phenol-chloroform protocol^67^. DNA libraries were constructed according to the manufacturer’s instructions (Illumina) with 350 bp insertions. Whole genome sequencing was carried out on the Illumina Novaseq. 6000 platform with a paired-end read length of 150 bp at Berry Genomics company (Beijing, China). The average sequencing depth per individual was 15 folds. Adapter sequencing, low-quality reads were filtered to obtain clean data. We further added sequences obtained from GenBank including more sampling localities (the Netherland, Canada, Iran, Kazakhstan, Mongolia) for investigation (Table S3)^21,68,69^ ^6,7,9^.

### Mitochondrial sequence assembly and gene annotation

Complete mitogenomes were assembled using the MITOBim 1.8 pipeline^70^, which relies on the baiting and iterative mapping strategy implemented in MIRA4^71^. BLASTn was used to determine the accuracy of the assembled mitogenomes by comparison with the complete mitogenomes of the Collared Flycatcher (*Ficedula albicollis*) (NC_021621.1) and Isabelline Wheatear (*O. isabellina*) (NC_040290.1). Protein coding genes, tRNAs and rRNAs were annotated by the MITOS WebServer (http://mitos2.bioinf.uni-leipzig.de/index.py) and blasted against the NCBI database of mitochondrial sequences. The PCG sequences were extracted and concatenated by custom python scripts. tRNA prediction was further performed by tRNAscan-SE v1.3.1^72^. Secondary structures of 16S and 12S RNA were derived using the RNAfold WebServer (http://rna.tbi.univie.ac.at/cgi-bin/RNAWebSuite/RNAfold.cgi) with minimum free energy (MFE) and partition function. Mitochondrial maps were generated by CGView^73^. As seen in the chicken *Gallus gallus*^32^ and the outgroup *O. isabellina*^66^, the highly variable control regions (CR) of all investigated *Oenanthe* spp. were located between the tRNA-Phe and tRNA-Glu. Since our mitogenome alignments were derived from next-generation sequencing data, coverage became rapidly reduced in the CR. We therefore excluded the CR from downstream phylogeographical analyses to avoid incomplete assemblies or ambiguous alignments.

### Phylogenetic analysis

PCG sequences were aligned by MAFFT v7.037^74^. Neither indels nor interal stop codons were detected in the alignments. SequenceMatrix v.1.7.8^75^ was applied to concatenate the alignments for different gene sets. PartitionFinder v.2.1.1^76^ was applied to determine the best partition arrangement and optimal substitution models of sequence evolution for downstream phylogenetic analyses. Four datasets were pre-defined in the input configuration files (1) 13 PCGs, two rRNAs and combined 22 tRNAs with 15529 residues, (2) 13 PCGs with gene partition, (3) 13 PCGs with codon partition and (4) each single gene with or without partition. The greedy algorithm with unlinked branch lengths estimation and Akaike information criterion with correction (AICc) were used to search for appropriate partitions schemes^77^.

Phylogenetic trees were constructed based on the best-fit partition schemes suggested by PartitionFinder. Bayesian inference (BI) analysis was applied by MrBayes version 3.2.2^78^. The datasets were run with 2 simultaneous Markov Chain Monte Carlo (MCMC) runs on one cold and three heated chains to confirm the convergence of posterior probability distributions^79^. Analyses were set to run for 10 million generations with sampling conducted every 1,000 generations until stationarity was reached, i.e., average standard deviation of split frequencies less than 0.01. The initial 25% of the total trees were discarded as burn-in. The speciation time was estimated with the GTR substitution model implemented in BEAST 1.8.3^80–82^. We tested a Yule speciation process with a strict clock model (2.1% per million years for *Cytb*) and estimated the mutation rate for the remaining markers^83^. All the analyses were performed in the CIPRES Science Gateway (http://www.phylo.org) and visualization was achieved by FigTree version 1.4.1 (http://beast.bio.ed.ac.uk/FigTree). Population genetic analyses, neutrality tests and mismatch distributions were calculated in ARLEQUIN version 3.5.1.3^84^ and DnaSP version 5.1^85^.

## Supplementary information

Supplementary Information.
