# Supplementary material for: Can Mitogenomes of the Northern Wheatear (Oenanthe oenanthe) Reconstruct Its Phylogeography and Reveal the Origin of Migrant Birds?
Source: Sci Rep. 2020 Jun 9;10:9290. doi: 10.1038/s41598-020-66287-0 (PMC7283232; doi:10.1038/s41598-020-66287-0)
Supplement: Supplementary file 1 — Supplementary Information. [file 41598_2020_66287_MOESM1_ESM.pdf]

Can Mitogenomes of the Northern Wheatear (*Oenanthe oenanthe*) Reconstruct Its Phylogeography and Reveal the Origin of Migrant Birds?

Erjia Wang [1, *], Dezhi Zhang [2,3], Markus Santhosh Braun [1], Agnes Hotz-Wagenblatt [4], Tomas Pärt [5], Debora Arlt[5], Heiko Schmaljohann[6,7], Franz Bairlein [6], Fumin Lei [2,3,8] and Michael Wink [1, *]

1. Institute of Pharmacy and Molecular Biotechnology, Heidelberg University, Heidelberg, Germany
2. Key laboratory of Zoological Systematics and Evolution, Institute of Zoology, Chinese Academy of Sciences, Beijing, China
3. College of Life Sciences, University of Chinese Academy of Sciences, Beijing, China
4. Omics IT and Data Management Core Facility, German Cancer Research Center, Heidelberg University, Heidelberg, Germany
5. Department of Ecology, Swedish University of Agricultural Science, Uppsala, Sweden
6. Institute of Avian Research "Vogelwarte Helgoland", Wilhelmshaven, Germany
7. Institute for Biology und Environmental Sciences (IBU), Carl von Ossietzky University of Oldenburg, Oldenburg, Germany
8. Center for Excellence in Animal Evolution and Genetics, Chinese Academy of Sciences, Kunming, China

* Correspondence: wang@uni-heidelberg.de (E.W.); wink@uni-heidelberg.de (M.W.)

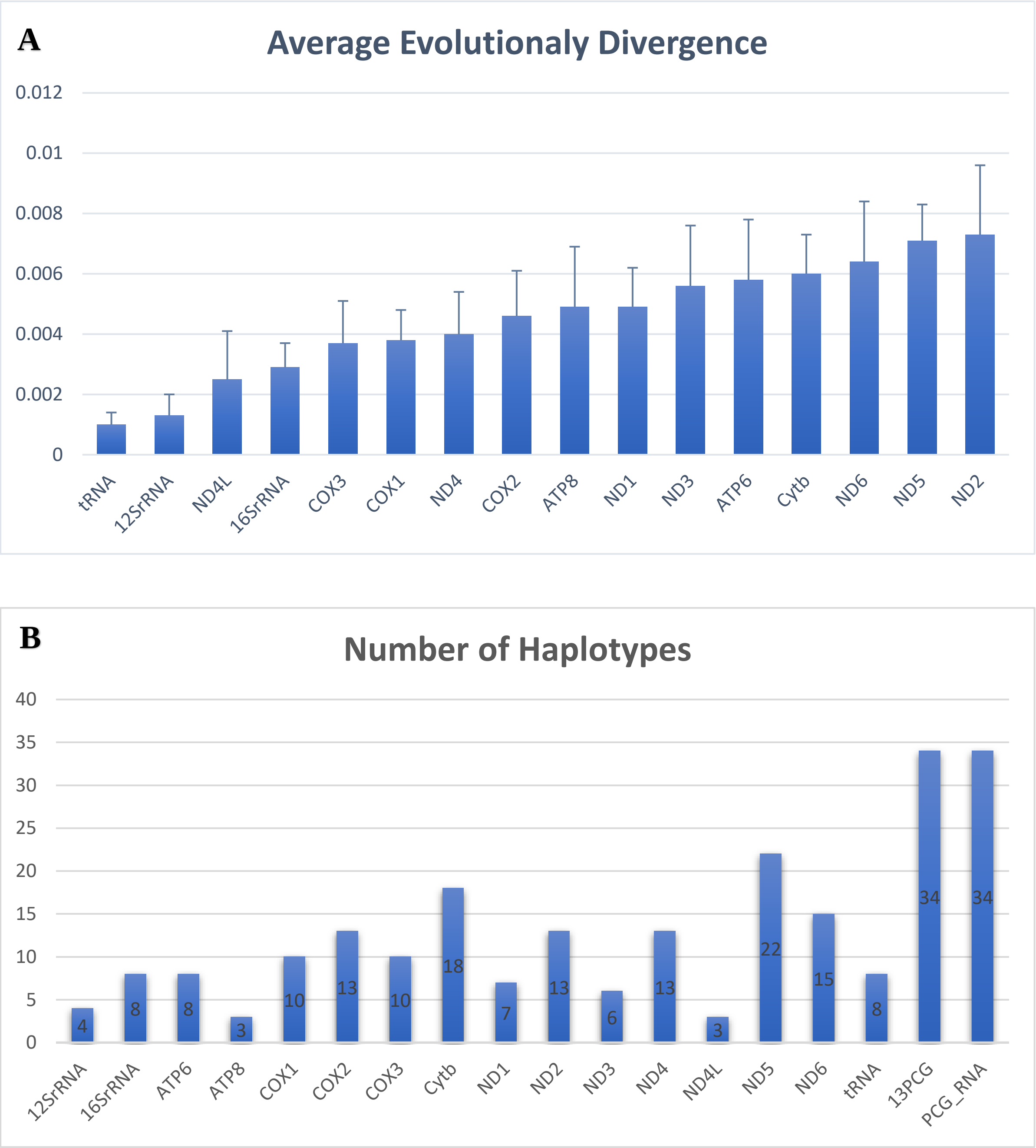

Figure S1 Characteristics of mtDNA markers. (A) Estimation of average evolutionary divergence over all sequence pairs for each marker. The calculations were generated the by the Maximum Composite Likelihood model in MEGA X. (B) Number of haplotypes for each marker for 38 breeding samples.

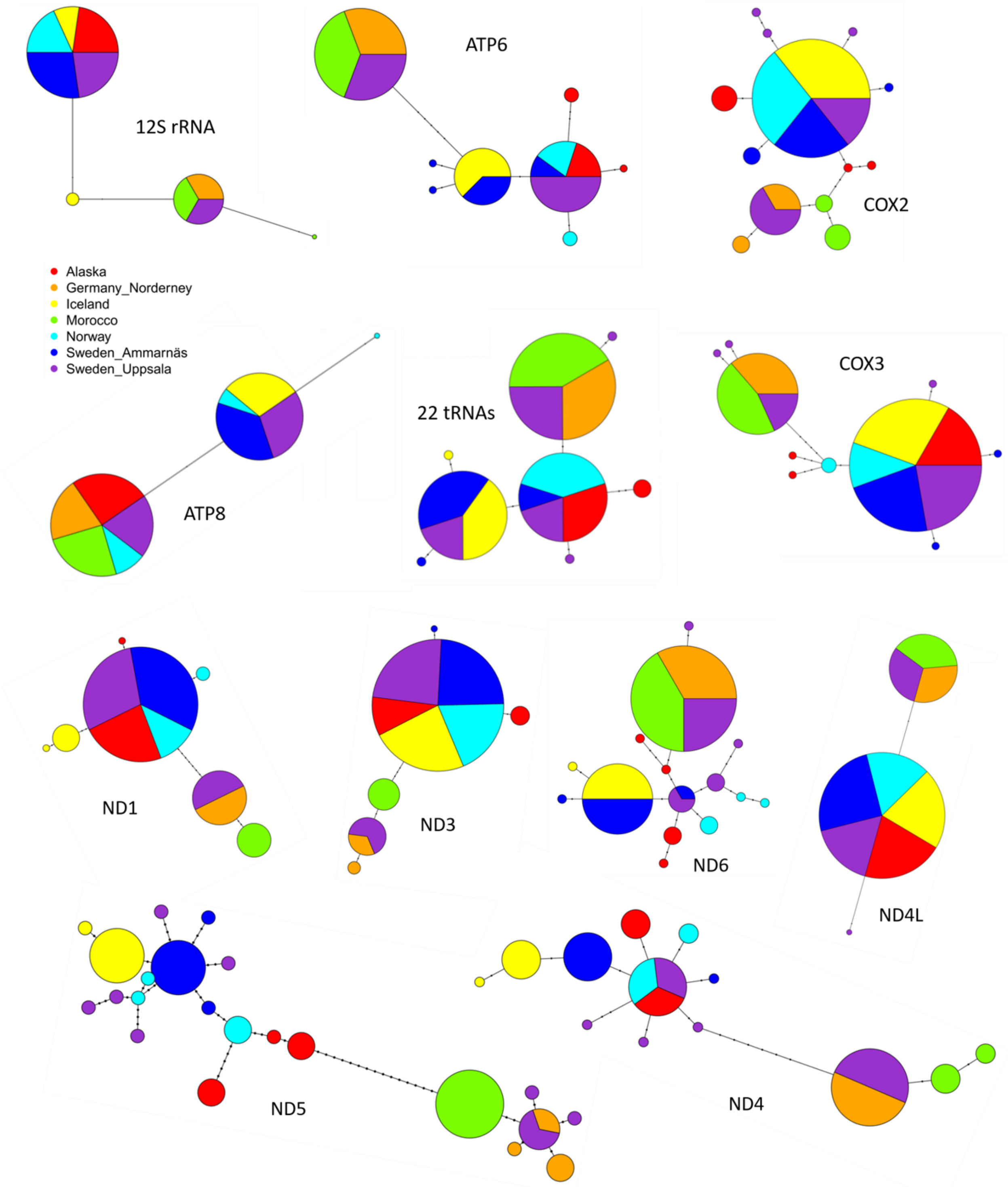

Figure S2 Haplotype network of single mitochondrial markers for 38 individuals of *Oenanthe*. Size of the circles represents the frequency of haplotypes. The breeding areas are coded by the colors. Each dot indicates one mutation step. Samples from Morocco are black throat wheatears (*O. seebohmi*); samples from Iceland are Greenland wheatears (*O. o. leucorhoa*); samples from Alaska, Germany, Norway and Sweden are nominate wheatears (*O. o. oenanthe*). The classification of (sub)species are identified by the morphological data.

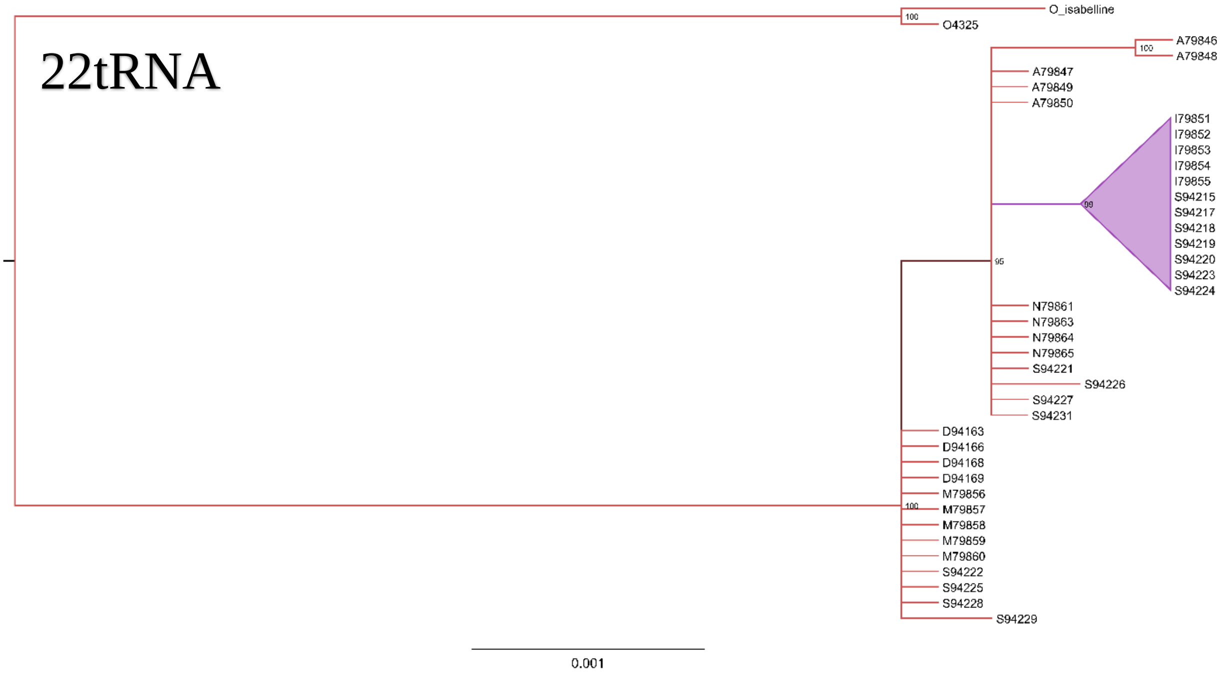
22tRNA
O_isabelline
O4325
A79846
A79848
A79847
A79849
A79850
I79851
I79852
I79853
I79854
I79855
S94215
S94217
S94218
S94219
S94220
S94223
S94224
N79861
N79863
N79864
N79865
S94221
S94226
S94227
S94231
D94163
D94166
D94168
D94169
M79856
M79857
M79858
M79859
M79860
S94222
S94225
S94228
S94229
0.001

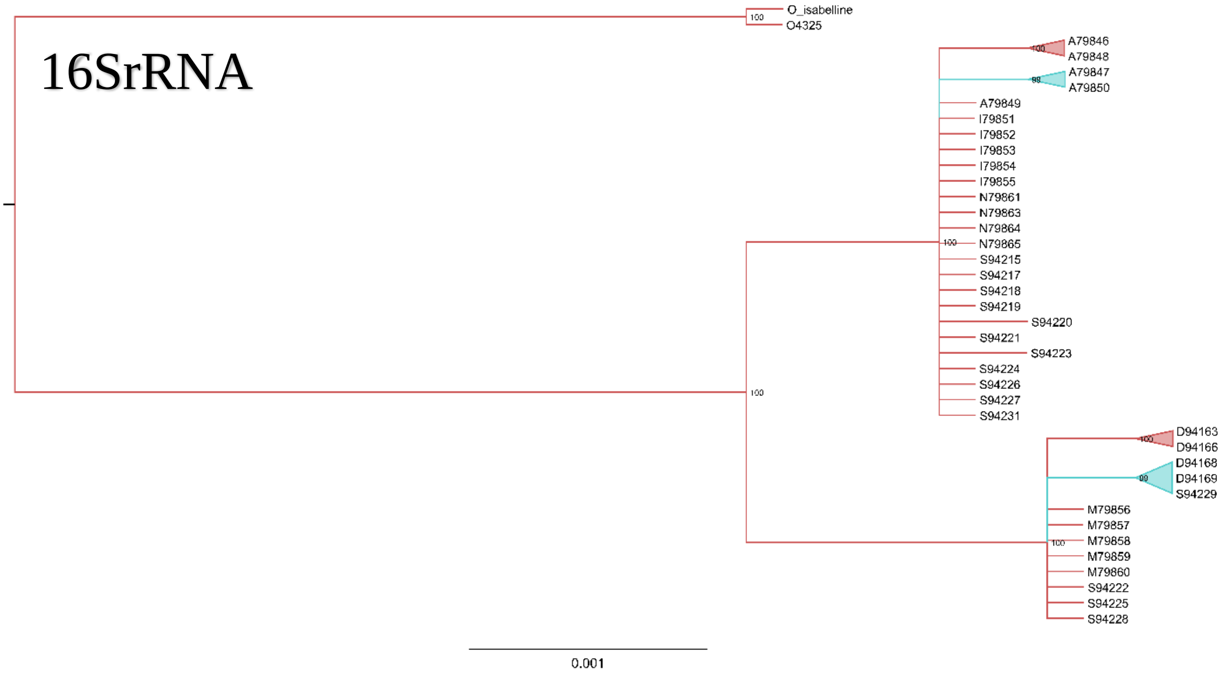
16SrRNA
O_isabelline
O4325
A79846
A79848
A79847
A79850
A79849
I79851
I79852
I79853
I79854
I79855
N79861
N79863
N79864
N79865
S94215
S94217
S94218
S94219
S94220
S94221
S94223
S94224
S94226
S94227
S94231
D94163
D94166
D94168
D94169
S94229
M79856
M79857
M79858
M79859
M79860
S94222
S94225
S94228
0.001

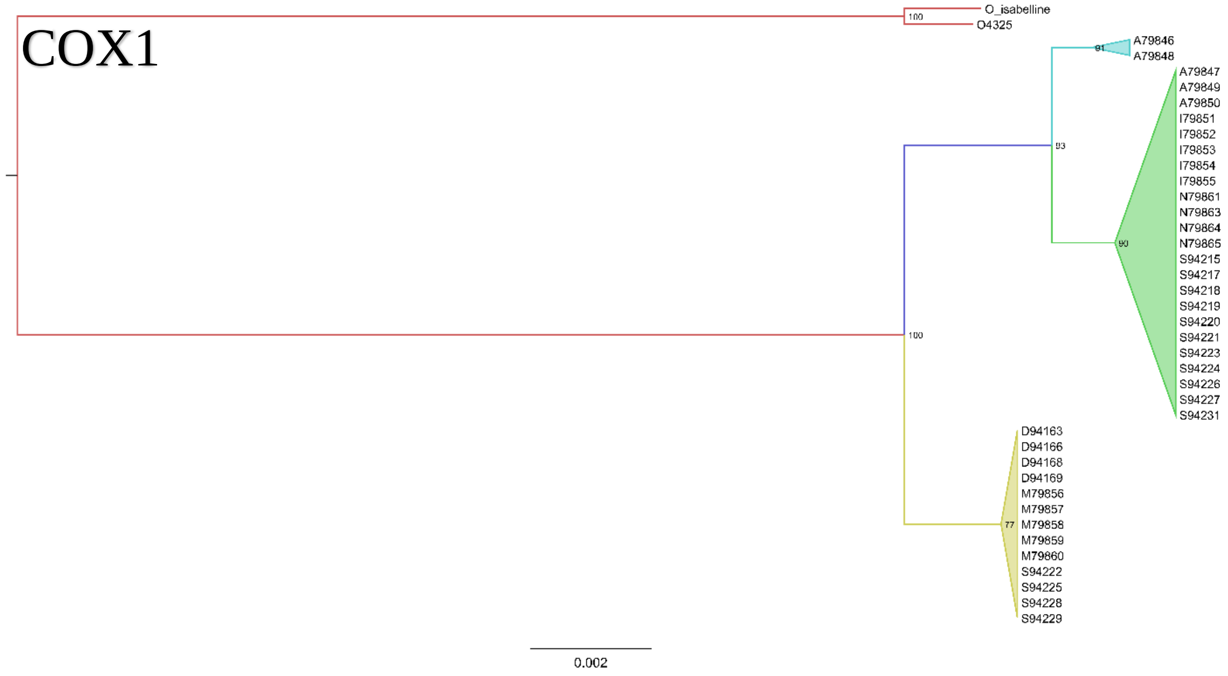
COX1
O_isabelline
O4325
A79846
A79848
A79847
A79849
A79850
I79851
I79852
I79853
I79854
I79855
N79861
N79863
N79864
N79865
S94215
S94217
S94218
S94219
S94220
S94221
S94223
S94224
S94226
S94227
S94231
D94163
D94166
D94168
D94169
M79856
M79857
M79858
M79859
M79860
S94222
S94225
S94228
S94229
0.002

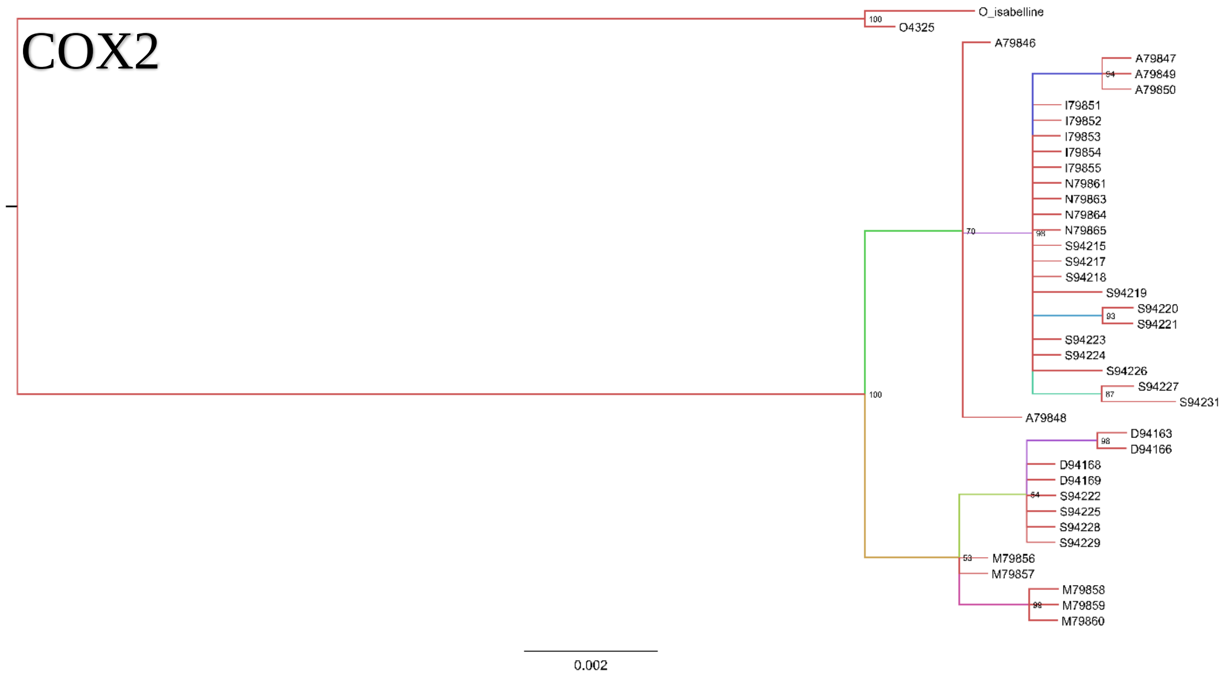
COX2
O_isabelline
O4325
A79846
A79847
A79849
A79850
I79851
I79852
I79853
I79854
I79855
N79861
N79863
N79864
N79865
S94215
S94217
S94218
S94219
S94220
S94221
S94223
S94224
S94226
S94227
S94231
A79848
D94163
D94166
D94168
D94169
S94222
S94225
S94228
S94229
M79856
M79857
M79858
M79859
M79860
0.002

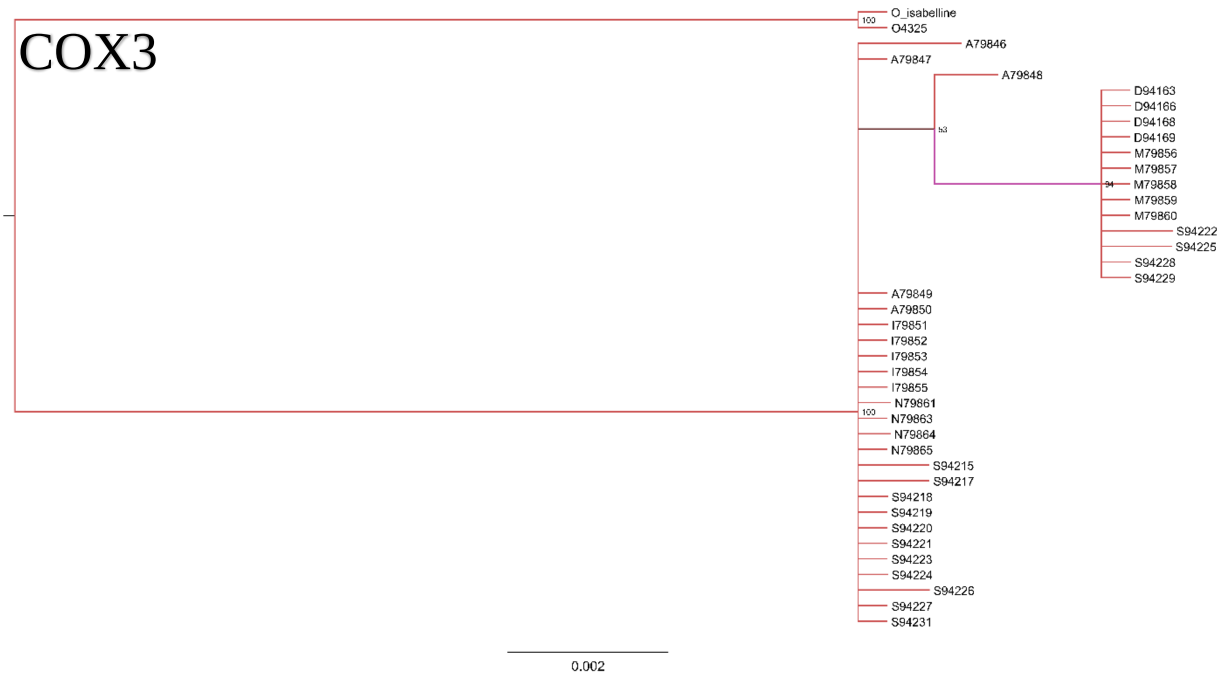
COX3
O_isabelline
100
O4325
A79846
A79847
A79848
D94163
D94166
D94168
D94169
M79856
M79857
M79858
M79859
M79860
S94222
S94225
S94228
S94229
A79849
A79850
I79851
I79852
I79853
I79854
I79855
N79861
100
N79863
N79864
N79865
S94215
S94217
S94218
S94219
S94220
S94221
S94223
S94224
S94226
S94227
S94231
0.002

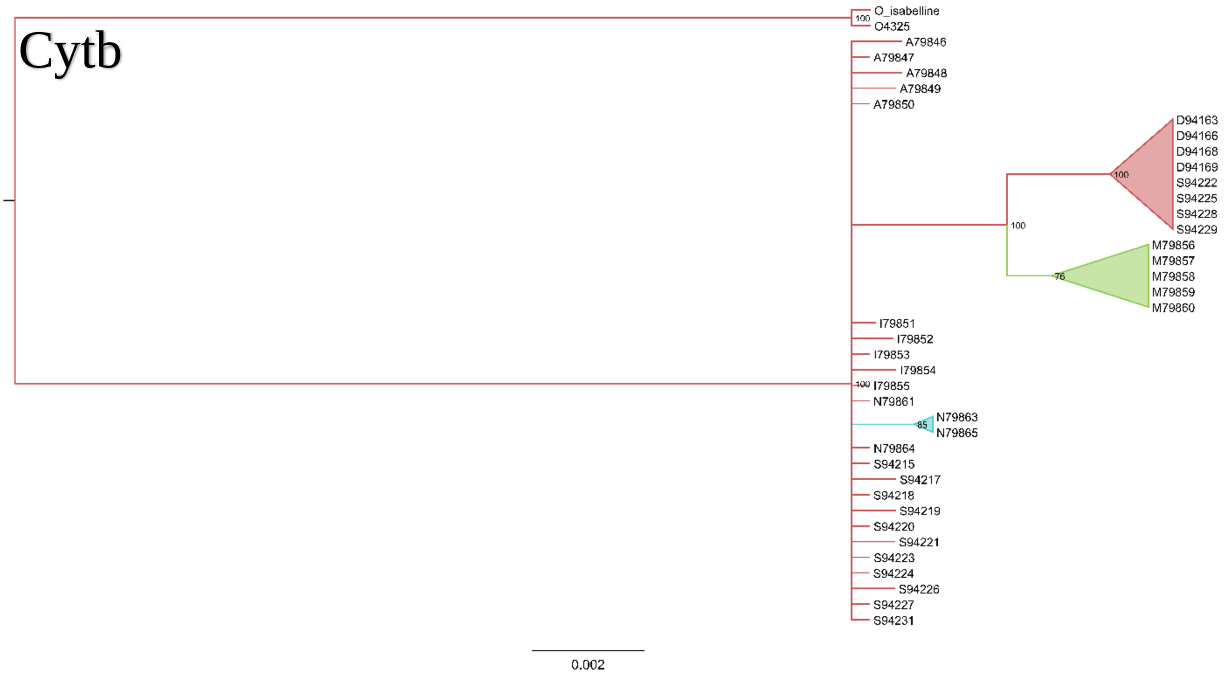
Cytb
O_isabelline
100
O4325
A79846
A79847
A79848
A79849
A79850
D94163
D94166
D94168
D94169
S94222
S94225
S94228
S94229
100
100
M79856
M79857
M79858
M79859
M79860
76
I79851
I79852
I79853
I79854
100
I79855
N79861
N79863
N79865
85
N79864
S94215
S94217
S94218
S94219
S94220
S94221
S94223
S94224
S94226
S94227
S94231
0.002

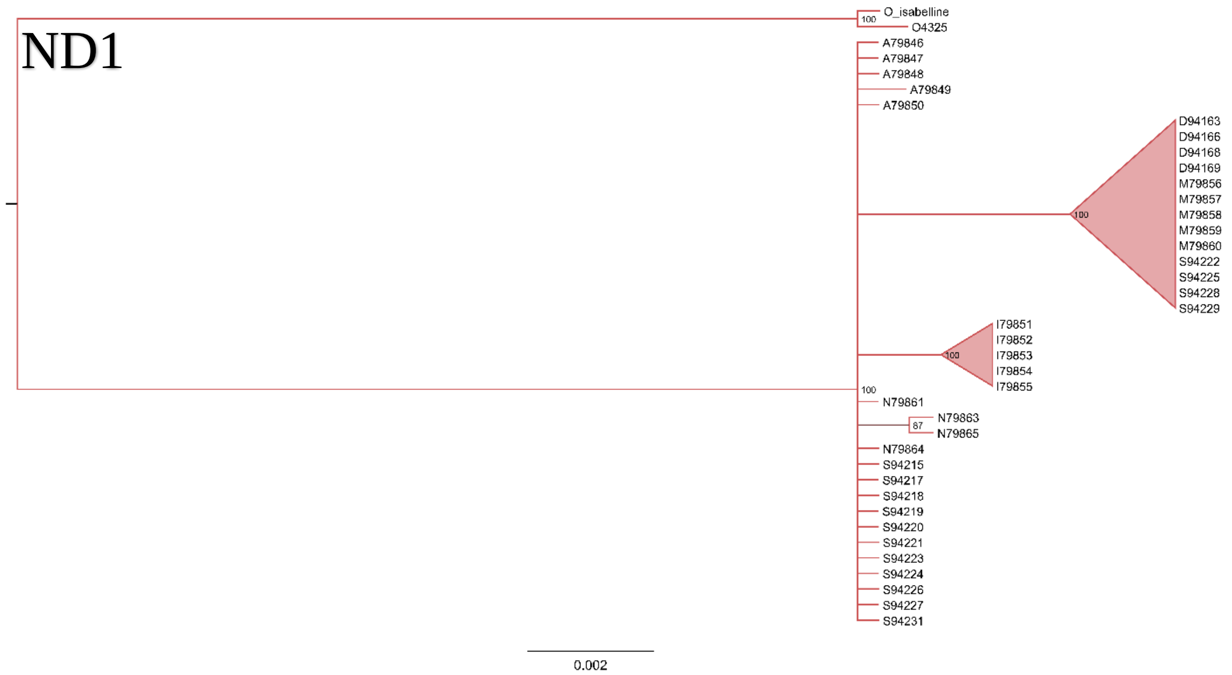
ND1
O_isabelline
O4325
100
A79846
A79847
A79848
A79849
A79850
D94163
D94166
D94168
D94169
M79856
M79857
M79858
M79859
M79860
S94222
S94225
S94228
S94229
100
I79851
I79852
I79853
I79854
I79855
100
100
N79861
N79863
N79865
87
N79864
S94215
S94217
S94218
S94219
S94220
S94221
S94223
S94224
S94226
S94227
S94231
0.002

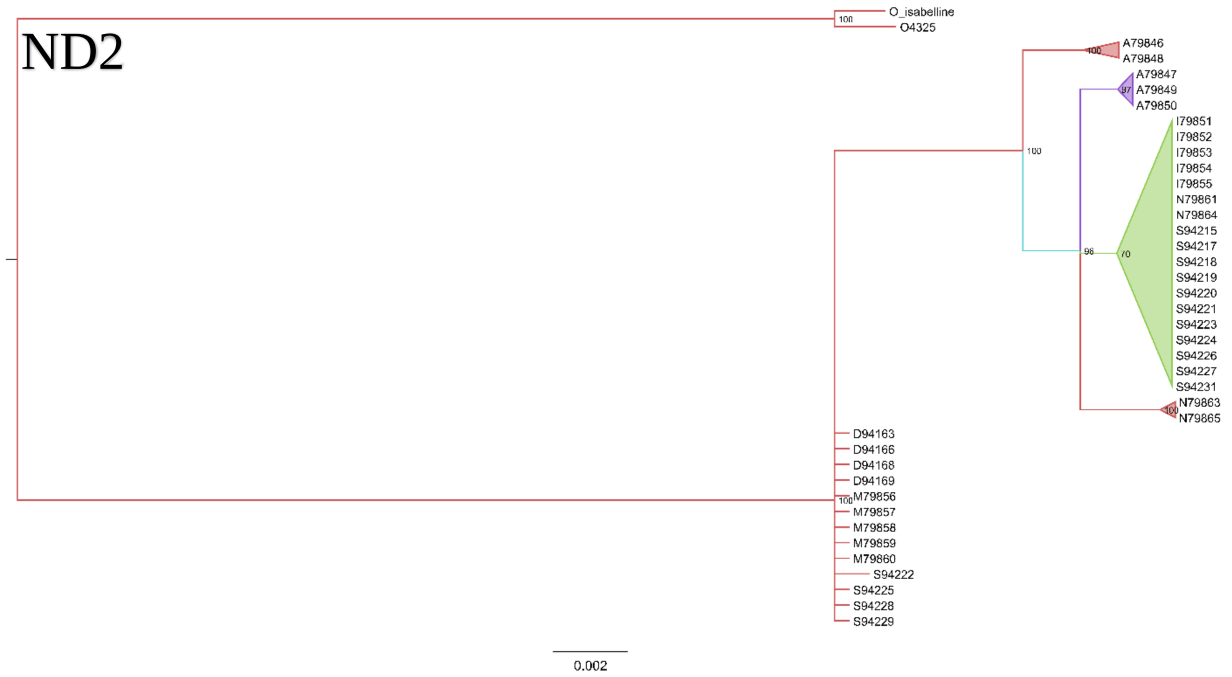
ND2
O_isabelline
O4325
100
A79846
A79848
100
A79847
A79849
A79850
97
I79851
I79852
I79853
I79854
I79855
N79861
N79864
S94215
S94217
S94218
S94219
S94220
S94221
S94223
S94224
S94226
S94227
S94231
100
98
70
N79863
N79865
100
D94163
D94166
D94168
D94169
M79856
M79857
M79858
M79859
M79860
S94222
S94225
S94228
S94229
100
0.002

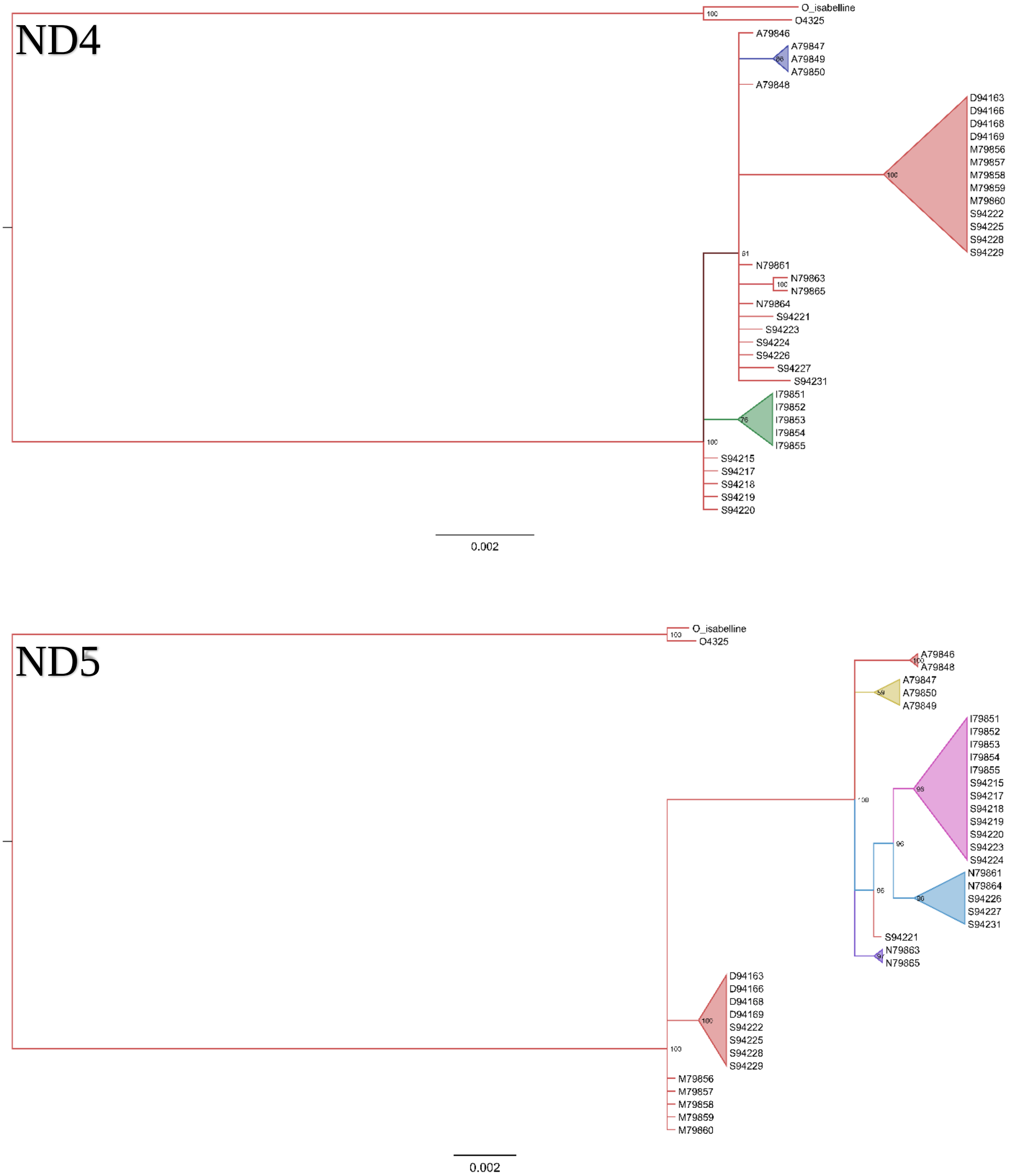

Figure S3. MrBayes reconstruction of the *Oenanthe oenanthe* and its outgroup *Oenanthe isabellina*. Numbers above nodes refer to the support values of Bayesian posterior probability. Sample names indicate the localities by the capital letter, ‘A’ refers to Alaska; ‘D’ refers to Germany; ‘I’ refers to Iceland; ‘M’ refers to Morocco; ‘N’ refers to Norway and ‘S’ refers to Sweden. Samples from Morocco are black throat wheatears (*O. seebohmi*); samples from Iceland are Greenland wheatears (*O. o. leucorhoa*); samples from Alaska, Germany, Norway and Sweden are nominate wheatears (*O. o. oenanthe*).

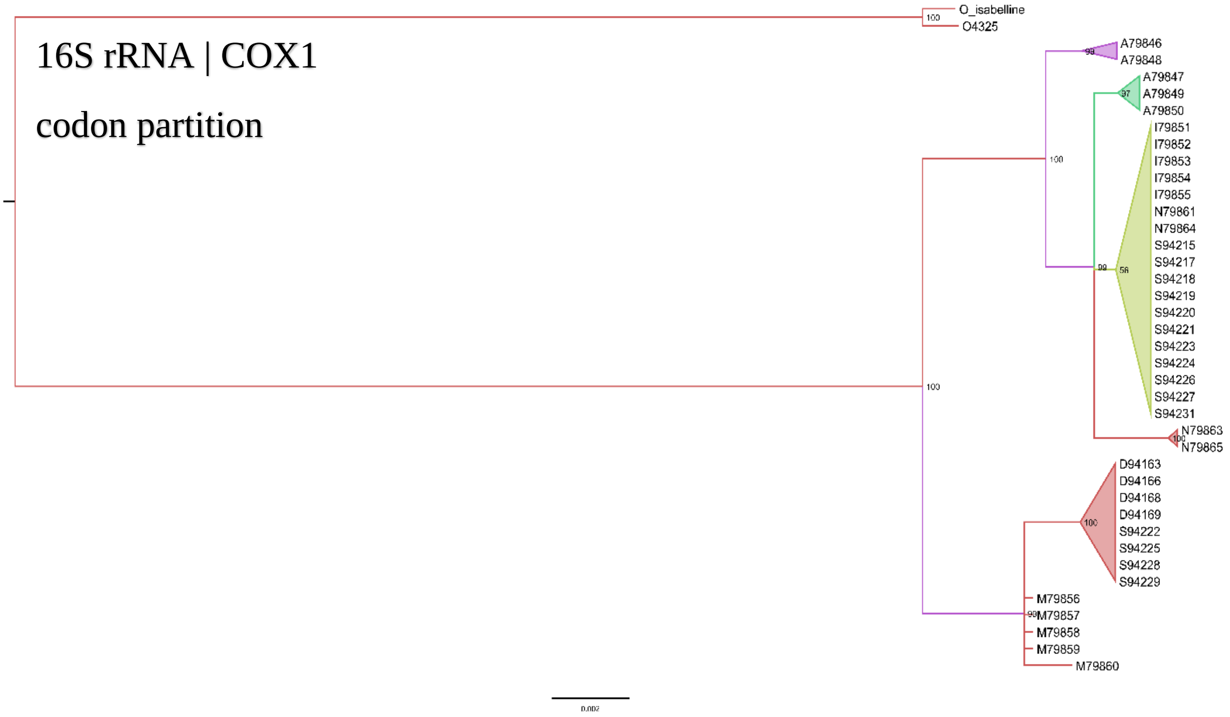
16S rRNA | COX1
codon partition
O_isabelline
O4325
A79846
A79848
A79847
A79849
A79850
I79851
I79852
I79853
I79854
I79855
N79861
N79864
S94215
S94217
S94218
S94219
S94220
S94221
S94223
S94224
S94226
S94227
S94231
N79863
N79865
D94163
D94166
D94168
D94169
S94222
S94225
S94228
S94229
M79856
M79857
M79858
M79859
M79860

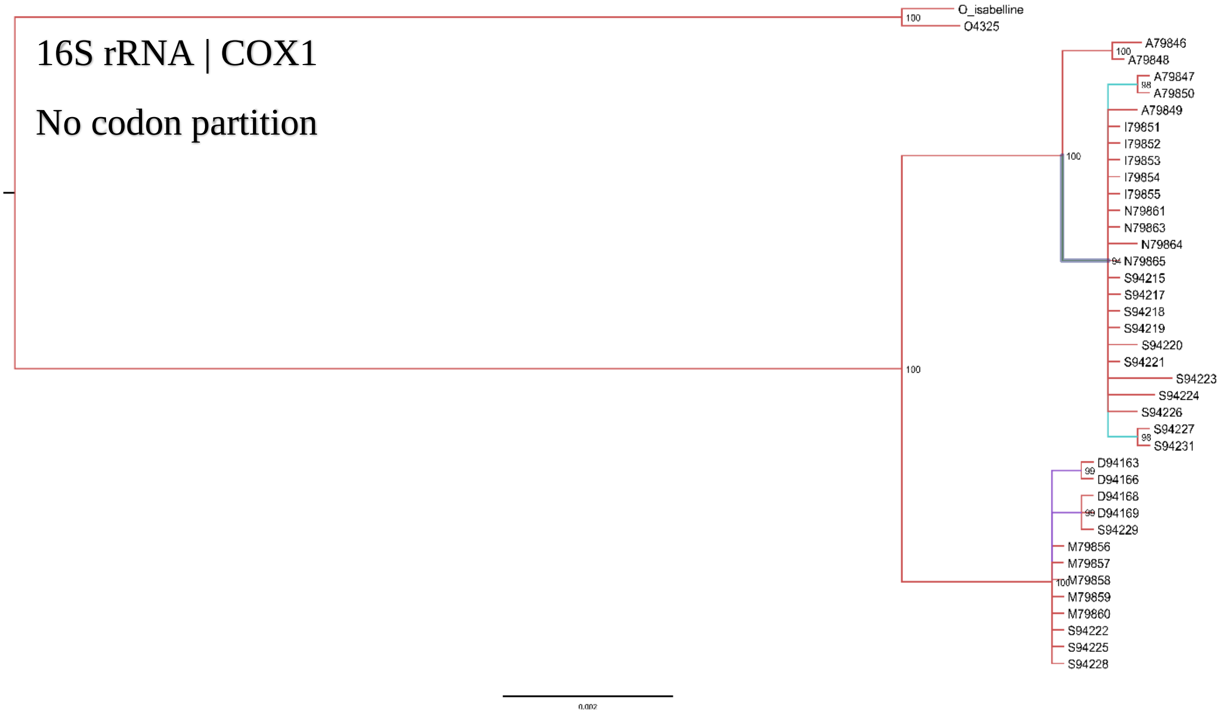
16S rRNA | COX1
No codon partition
O_isabelline
O4325
A79846
A79848
A79847
A79850
A79849
I79851
I79852
I79853
I79854
I79855
N79861
N79863
N79864
N79865
S94215
S94217
S94218
S94219
S94220
S94221
S94223
S94224
S94226
S94227
S94231
D94163
D94166
D94168
D94169
S94229
M79856
M79857
M79858
M79859
M79860
S94222
S94225
S94228

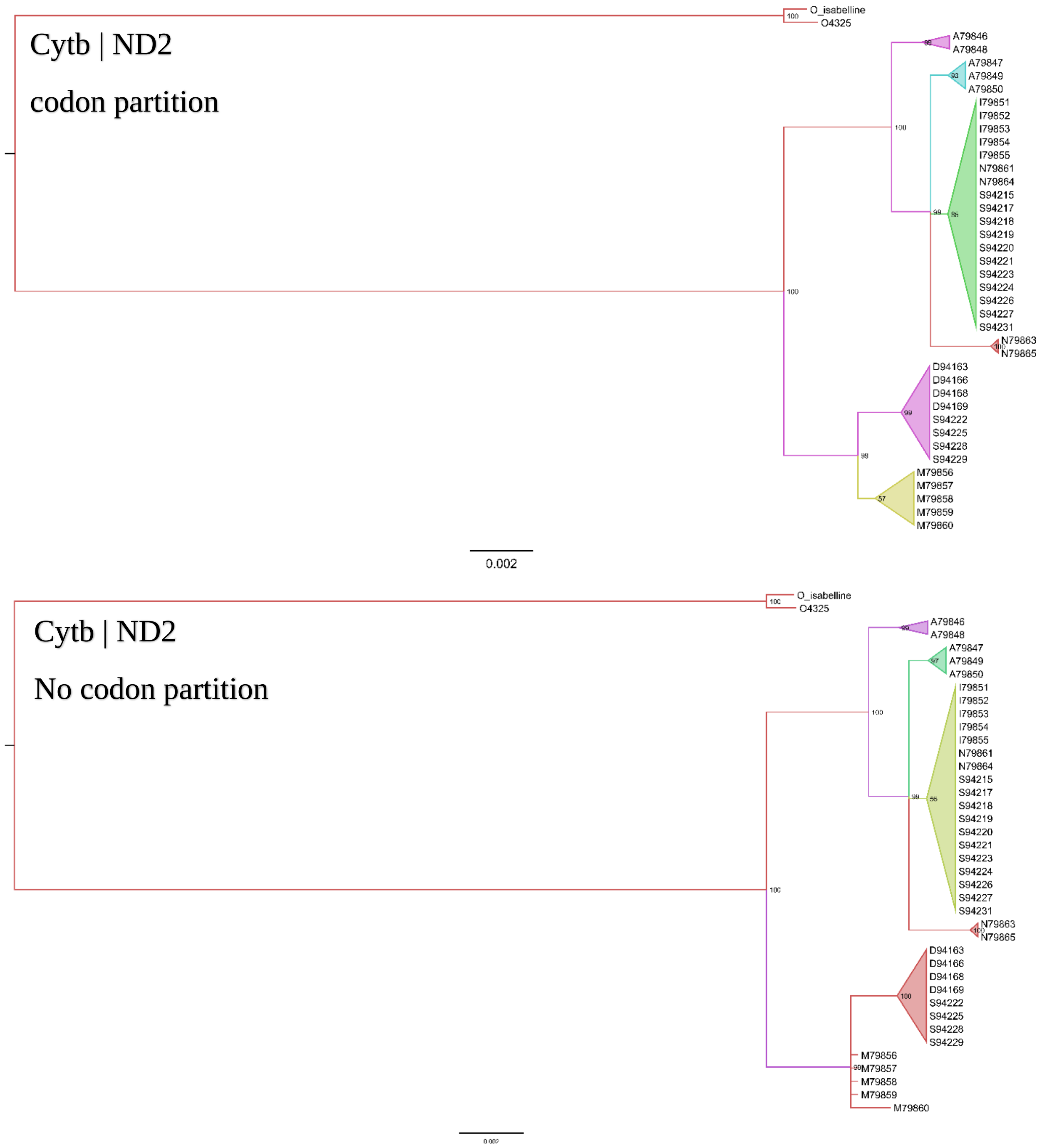

Figure S4. MrBayes reconstruction of the *Oenanthe oenanthe* and its outgroup *Oenanthe isabellina*. Numbers above nodes refer to the support values of Bayesian posterior probability. Sample names indicate the localities by the capital letter, 'A' refers to Alaska; 'D' refers to Germany; 'I' refers to Iceland; 'M' refers to Morocco; 'N' refers to Norway and 'S' refers to Sweden. Samples from Morocco are Seebohmi Wheatears (*O. seebohmi*); samples from Iceland are Greenland wheatears (*O. o. leucorhoa*); samples from Alaska, Germany, Norway and Sweden are nominate wheatears (*O. o. oenanthe*).

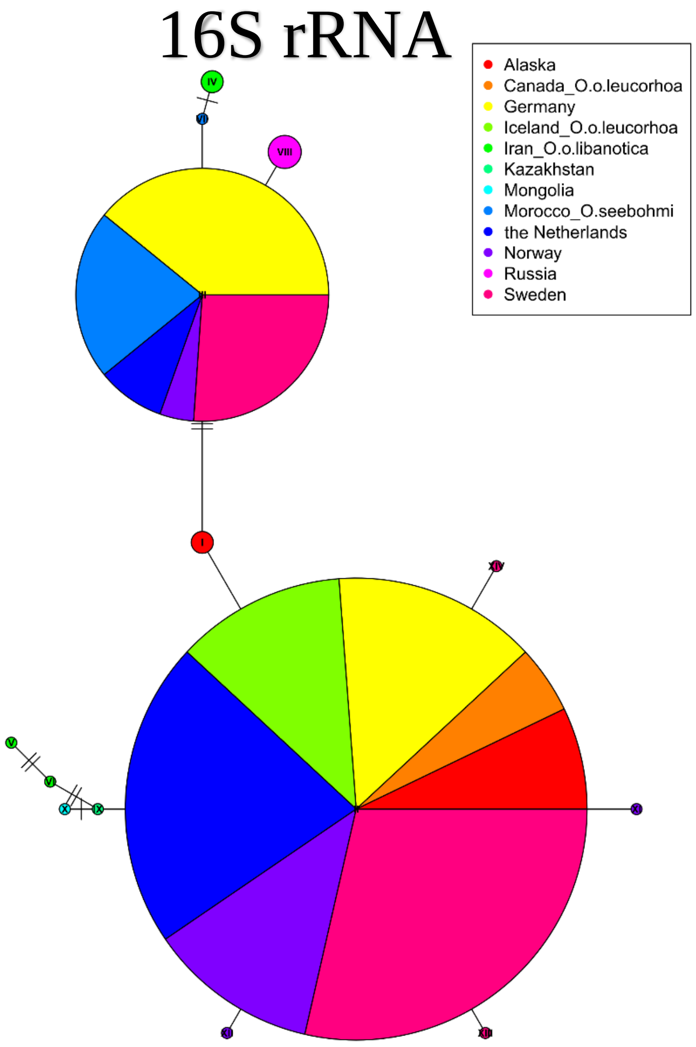

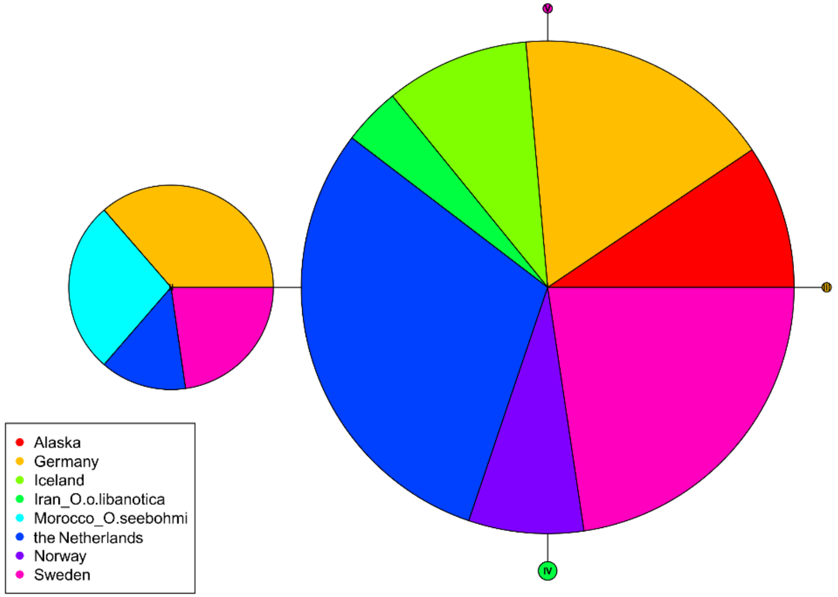

Figure S5. Haplotype network for *Oenanthe* including the samples from previous studies. Sample information are shown in the TableS1 and TableS2. Size of the circles represents the frequency of haplotypes. The sample localities are coded by the colors. Each dot indicates one mutation step. Samples from Morocco are Seebohmi Wheatears (*Oenanthe seebohmi*); samples from Iceland and Canada are Greenland Wheatears (*O. o. leucorhoa*); samples from Iran belong to *O. o. libanotica*. The rest of the samples are nominate wheatears (*O. o. oenanthe*). The classification of (sub)species are identified by the morphological data.

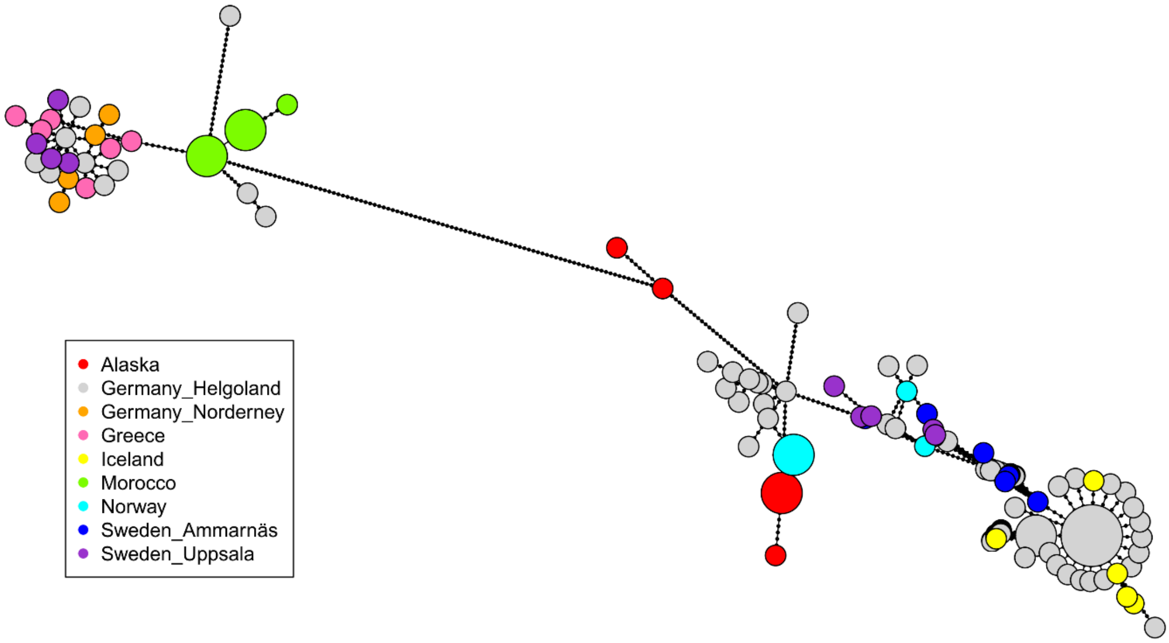

Figure S6. Haplotype network for 117 individuals of *Oenanthe* based on 13 PCGs, 14 tRNAs and 2 rRNAs. Size of the circles represents the frequency of haplotypes. The breeding areas are coded by the colors. Each dot indicates one mutation step. Samples from Morocco are Seebohmi Wheatears (*O. seebohmi*); samples from Iceland are Greenland wheatears (*O. o. leucorhoa*); samples from Alaska, Germany, Norway and Sweden are nominate wheatears (*O. o. oenanthe*); samples from Helgoland and Greece are migrating birds. The classification of (sub)species are identified by the morphological data.

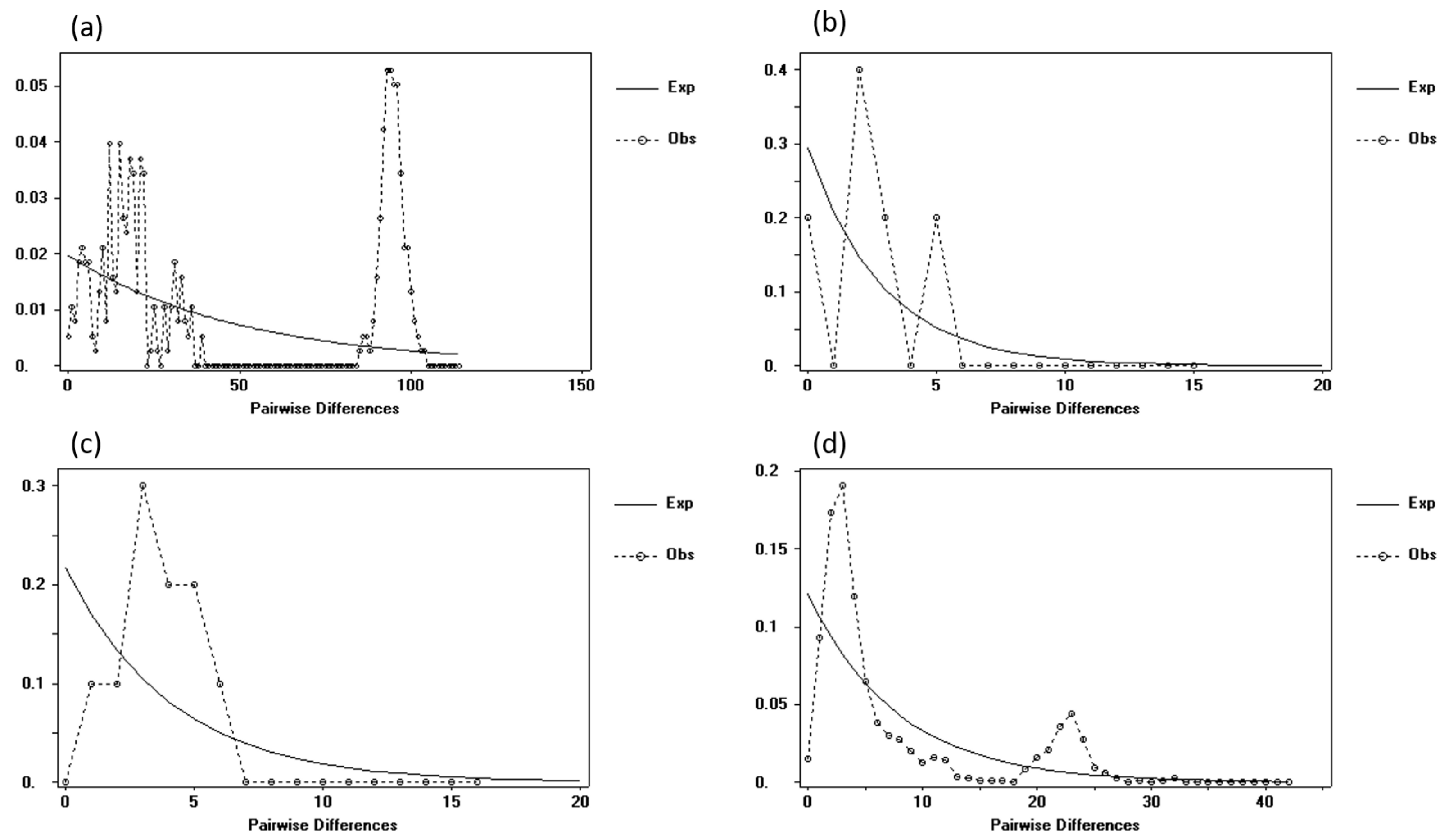

Figure S7. Mismatch distribution under the demographic expansion model. The graphs illustrate the frequency of pairwise difference among (a) breeding populations of *O.o.oenanthe*; (b) Morocco population of *O.seebohmi*; (c) Iceland population of *O. o. leucorhoa* and (d) migrating and breeding *O. o. leucorhoa*. based on 13 PCG. We included the migrating *O. o. leucorhoa* for the calculation, because of the limited sample size of breeding population.

Table S1 Mitochondrial molecular indices of 13 PCG

taxa	population	sample size	No. of polymorphic sites	haplotype diversity	nucleotide diversity	Tajima's D	P values	Fu's	P values
O.o.oenanthe	Alaska	5	39	0.9000 ± 0.1610	0.1010 ± 0.0631	0.7549	0.7560	2.8224	0.8650
O.o.oenanthe	Germany	4	6	1.0000 ± 0.1768	0.0180 ± 0.0137	1.1680	0.8350	-0.9463	0.1140
O.o.oenanthe	Norway	4	22	0.8333 ± 0.2224	0.0679 ± 0.0463	1.5698	0.9190	3.1779	0.8790
O.o.oenanthe	Ammarnäs	6	24	1.0000 ± 0.0962	0.0402 ± 0.0251	-1.3831	0.0560	-1.2713	0.1440
O.o.oenanthe	Uppsala	9	124	1.0000 ± 0.0524	0.2879 ± 0.1561	1.4859	0.9520	0.2284	0.3230
O.o.leucorhoa	Iceland	5	9	1.0000 ± 0.1265	0.0177 ± 0.0126	-1.1844	0.0310	-1.9011	0.0550
O.seebohmi	Morocco	5	5	0.8000 ± 0.1640	0.0118 ± 0.0090	0.0000	0.5780	0.9518	0.6350

Table S2 Sample List

Species	IPMB	Locality	Country	Institute of Collector	GenBank	Tissue	Status
Oenanthe oenanthe	A79846	Alaska	United States	Vogelwarte Helgoland, Wilhelmshaven	MT430990	blood	Breeding
Oenanthe oenanthe	A79847	Alaska	United States	Vogelwarte Helgoland, Wilhelmshaven	MT430991	blood	Breeding
Oenanthe oenanthe	79848	Alaska	United States	Vogelwarte Helgoland, Wilhelmshaven	MT430992	blood	Breeding
Oenanthe oenanthe	A79849	Alaska	United States	Vogelwarte Helgoland, Wilhelmshaven	MT430993	blood	Breeding
Oenanthe oenanthe	A79850	Alaska	United States	Vogelwarte Helgoland, Wilhelmshaven	MT430994	blood	Breeding
Oenanthe oenanthe	I79851		Iceland	Vogelwarte Helgoland, Wilhelmshaven	MT430995	blood	Breeding
Oenanthe oenanthe	I79852		Iceland	Vogelwarte Helgoland, Wilhelmshaven	MT430996	blood	Breeding
Oenanthe oenanthe	I79853		Iceland	Vogelwarte Helgoland, Wilhelmshaven	MT430997	blood	Breeding
Oenanthe oenanthe	I79854		Iceland	Vogelwarte Helgoland, Wilhelmshaven	MT430998	blood	Breeding
Oenanthe oenanthe	I79855		Iceland	Vogelwarte Helgoland, Wilhelmshaven	MT430999	blood	Breeding
Oenanthe seebohmi	M79856		Morocco	Vogelwarte Helgoland, Wilhelmshaven	MT431000	blood	Breeding
Oenanthe seebohmi	M79857		Morocco	Vogelwarte Helgoland, Wilhelmshaven	MT431001	blood	Breeding
Oenanthe seebohmi	M79858		Morocco	Vogelwarte Helgoland, Wilhelmshaven	MT431002	blood	Breeding
Oenanthe seebohmi	M79859		Morocco	Vogelwarte Helgoland, Wilhelmshaven	MT431003	blood	Breeding
Oenanthe seebohmi	M79860		Morocco	Vogelwarte Helgoland, Wilhelmshaven	MT431004	blood	Breeding
Oenanthe oenanthe	N79861		Norway	Vogelwarte Helgoland, Wilhelmshaven	MT431005	blood	Breeding
Oenanthe oenanthe	N79863		Norway	Vogelwarte Helgoland, Wilhelmshaven	MT431006	blood	Breeding

Oenanthe oenanthe	N79864		Norway	Vogelwarte Helgoland, Wilhelmshaven	MT431007	blood	Breeding
Oenanthe oenanthe	N79865		Norway	Vogelwarte Helgoland, Wilhelmshaven	MT431008	blood	Breeding
Oenanthe oenanthe	D94163	Norderney	Germany	Vogelwarte Helgoland, Wilhelmshaven, Heiko Schmaljohann	MT431009	blood	Breeding
Oenanthe oenanthe	D94166	Norderney	Germany	Vogelwarte Helgoland, Wilhelmshaven, Heiko Schmaljohann	MT431010	blood	Breeding
Oenanthe oenanthe	D94168	Norderney	Germany	Vogelwarte Helgoland, Wilhelmshaven, Heiko Schmaljohann	MT431011	blood	Breeding
Oenanthe oenanthe	D94169	Norderney	Germany	Vogelwarte Helgoland, Wilhelmshaven, Heiko Schmaljohann	MT431012	blood	Breeding
Oenanthe oenanthe	S94215	Ammarnäs	Sweden	Department of Ecology, Swedish University of Agricultural Science	MT431013	blood	Breeding
Oenanthe oenanthe	S94217	Ammarnäs	Sweden	Department of Ecology, Swedish University of Agricultural Science	MT431014	blood	Breeding
Oenanthe oenanthe	S94218	Ammarnäs	Sweden	Department of Ecology, Swedish University of Agricultural Science	MT431015	blood	Breeding
Oenanthe oenanthe	S94219	Ammarnäs	Sweden	Department of Ecology, Swedish University of Agricultural Science	MT431016	blood	Breeding
Oenanthe oenanthe	S94220	Ammarnäs	Sweden	Department of Ecology, Swedish University of Agricultural Science	MT431017	blood	Breeding
Oenanthe oenanthe	S94221	Ammarnäs	Sweden	Department of Ecology, Swedish University of Agricultural Science	MT431018	blood	Breeding
Oenanthe oenanthe	S94222	Uppsala	Sweden	Department of Ecology, Swedish University of Agricultural Science	MT431019	blood	Breeding
Oenanthe oenanthe	S94223	Uppsala	Sweden	Department of Ecology, Swedish University of Agricultural Science	MT431020	blood	Breeding
Oenanthe oenanthe	S94224	Uppsala	Sweden	Department of Ecology, Swedish University of Agricultural Science	MT431021	blood	Breeding
Oenanthe oenanthe	S94225	Uppsala	Sweden	Department of Ecology, Swedish University of Agricultural Science	MT431022	blood	Breeding
Oenanthe oenanthe	S94226	Uppsala	Sweden	Department of Ecology, Swedish University of Agricultural Science	MT431023	blood	Breeding
Oenanthe oenanthe	S94227	Uppsala	Sweden	Department of Ecology, Swedish University of Agricultural Science	MT431024	blood	Breeding
Oenanthe oenanthe	S94228	Uppsala	Sweden	Department of Ecology, Swedish University of Agricultural Science	MT431025	blood	Breeding

Oenanthe oenanthe	S94229	Uppsala	Sweden	Department of Ecology, Swedish University of Agricultural Science	MT431026	blood	Breeding
Oenanthe oenanthe	S94231	Uppsala	Sweden	Department of Ecology, Swedish University of Agricultural Science	MT431027	blood	Breeding
Oenanthe oenanthe	H93275	Helgoland	Germany	Vogelwarte Helgoland, Wilhelmshaven, Heiko Schmaljohann	MT431028	blood	Migrant
Oenanthe oenanthe	H93276	Helgoland	Germany	Vogelwarte Helgoland, Wilhelmshaven, Heiko Schmaljohann	MT431029	blood	Migrant
Oenanthe oenanthe	H93277	Helgoland	Germany	Vogelwarte Helgoland, Wilhelmshaven, Heiko Schmaljohann	MT431030	blood	Migrant
Oenanthe oenanthe	H93278	Helgoland	Germany	Vogelwarte Helgoland, Wilhelmshaven, Heiko Schmaljohann	MT431031	blood	Migrant
Oenanthe oenanthe	H93279	Helgoland	Germany	Vogelwarte Helgoland, Wilhelmshaven, Heiko Schmaljohann	MT431032	blood	Migrant
Oenanthe oenanthe	H93280	Helgoland	Germany	Vogelwarte Helgoland, Wilhelmshaven, Heiko Schmaljohann	MT431033	blood	Migrant
Oenanthe oenanthe	H93283	Helgoland	Germany	Vogelwarte Helgoland, Wilhelmshaven, Heiko Schmaljohann	MT431034	blood	Migrant
Oenanthe oenanthe	H93285	Helgoland	Germany	Vogelwarte Helgoland, Wilhelmshaven, Heiko Schmaljohann	MT431035	blood	Migrant
Oenanthe oenanthe	H93286	Helgoland	Germany	Vogelwarte Helgoland, Wilhelmshaven, Heiko Schmaljohann	MT431036	blood	Migrant
Oenanthe oenanthe	H93287	Helgoland	Germany	Vogelwarte Helgoland, Wilhelmshaven, Heiko Schmaljohann	MT431037	blood	Migrant
Oenanthe oenanthe	H93288	Helgoland	Germany	Vogelwarte Helgoland, Wilhelmshaven, Heiko Schmaljohann	MT431038	blood	Migrant
Oenanthe oenanthe	H93289	Helgoland	Germany	Vogelwarte Helgoland, Wilhelmshaven, Heiko Schmaljohann	MT431039	blood	Migrant
Oenanthe oenanthe	H93291	Helgoland	Germany	Vogelwarte Helgoland, Wilhelmshaven, Heiko Schmaljohann	MT431040	blood	Migrant
Oenanthe oenanthe	H93292	Helgoland	Germany	Vogelwarte Helgoland, Wilhelmshaven, Heiko Schmaljohann	MT431041	blood	Migrant
Oenanthe oenanthe	H93293	Helgoland	Germany	Vogelwarte Helgoland, Wilhelmshaven, Heiko Schmaljohann	MT431042	blood	Migrant
Oenanthe oenanthe	H93294	Helgoland	Germany	Vogelwarte Helgoland, Wilhelmshaven, Heiko Schmaljohann	MT431043	blood	Migrant
Oenanthe oenanthe	H93295	Helgoland	Germany	Vogelwarte Helgoland, Wilhelmshaven, Heiko Schmaljohann	MT431044	blood	Migrant

Oenanthe oenanthe	H93296	Helgoland	Germany	Vogelwarte Helgoland, Wilhelmshaven, Heiko Schmaljohann	MT431045	blood	Migrant
Oenanthe oenanthe	H93299	Helgoland	Germany	Vogelwarte Helgoland, Wilhelmshaven, Heiko Schmaljohann	MT431046	blood	Migrant
Oenanthe oenanthe	H93300	Helgoland	Germany	Vogelwarte Helgoland, Wilhelmshaven, Heiko Schmaljohann	MT431047	blood	Migrant
Oenanthe oenanthe	H93301	Helgoland	Germany	Vogelwarte Helgoland, Wilhelmshaven, Heiko Schmaljohann	MT431048	blood	Migrant
Oenanthe oenanthe	H93302	Helgoland	Germany	Vogelwarte Helgoland, Wilhelmshaven, Heiko Schmaljohann	MT431049	blood	Migrant
Oenanthe oenanthe	H93303	Helgoland	Germany	Vogelwarte Helgoland, Wilhelmshaven, Heiko Schmaljohann	MT431050	blood	Migrant
Oenanthe oenanthe	H93304	Helgoland	Germany	Vogelwarte Helgoland, Wilhelmshaven, Heiko Schmaljohann	MT431051	blood	Migrant
Oenanthe oenanthe	H93305	Helgoland	Germany	Vogelwarte Helgoland, Wilhelmshaven, Heiko Schmaljohann	MT431052	blood	Migrant
Oenanthe oenanthe	H93306	Helgoland	Germany	Vogelwarte Helgoland, Wilhelmshaven, Heiko Schmaljohann	MT431053	blood	Migrant
Oenanthe oenanthe	H93307	Helgoland	Germany	Vogelwarte Helgoland, Wilhelmshaven, Heiko Schmaljohann	MT431054	blood	Migrant
Oenanthe oenanthe	H93309	Helgoland	Germany	Vogelwarte Helgoland, Wilhelmshaven, Heiko Schmaljohann	MT431055	blood	Migrant
Oenanthe oenanthe	H93310	Helgoland	Germany	Vogelwarte Helgoland, Wilhelmshaven, Heiko Schmaljohann	MT431056	blood	Migrant
Oenanthe oenanthe	H93311	Helgoland	Germany	Vogelwarte Helgoland, Wilhelmshaven, Heiko Schmaljohann	MT431057	blood	Migrant
Oenanthe oenanthe	H93312	Helgoland	Germany	Vogelwarte Helgoland, Wilhelmshaven, Heiko Schmaljohann	MT431058	blood	Migrant
Oenanthe oenanthe	H93314	Helgoland	Germany	Vogelwarte Helgoland, Wilhelmshaven, Heiko Schmaljohann	MT431059	blood	Migrant
Oenanthe oenanthe	H93315	Helgoland	Germany	Vogelwarte Helgoland, Wilhelmshaven, Heiko Schmaljohann	MT431060	blood	Migrant
Oenanthe oenanthe	H93316	Helgoland	Germany	Vogelwarte Helgoland, Wilhelmshaven, Heiko Schmaljohann	MT431061	blood	Migrant
Oenanthe oenanthe	H93317	Helgoland	Germany	Vogelwarte Helgoland, Wilhelmshaven, Heiko Schmaljohann	MT431062	blood	Migrant
Oenanthe oenanthe	H93318	Helgoland	Germany	Vogelwarte Helgoland, Wilhelmshaven, Heiko Schmaljohann	MT431063	blood	Migrant

Oenanthe oenanthe	H93319	Helgoland	Germany	Vogelwarte Helgoland, Wilhelmshaven, Heiko Schmaljohann	MT431064	blood	Migrant
Oenanthe oenanthe	H93320	Helgoland	Germany	Vogelwarte Helgoland, Wilhelmshaven, Heiko Schmaljohann	MT431065	blood	Migrant
Oenanthe oenanthe	H93321	Helgoland	Germany	Vogelwarte Helgoland, Wilhelmshaven, Heiko Schmaljohann	MT431066	blood	Migrant
Oenanthe oenanthe	H93322	Helgoland	Germany	Vogelwarte Helgoland, Wilhelmshaven, Heiko Schmaljohann	MT431067	blood	Migrant
Oenanthe oenanthe	H93323	Helgoland	Germany	Vogelwarte Helgoland, Wilhelmshaven, Heiko Schmaljohann	MT431068	blood	Migrant
Oenanthe oenanthe	H93324	Helgoland	Germany	Vogelwarte Helgoland, Wilhelmshaven, Heiko Schmaljohann	MT431069	blood	Migrant
Oenanthe oenanthe	H93325	Helgoland	Germany	Vogelwarte Helgoland, Wilhelmshaven, Heiko Schmaljohann	MT431070	blood	Migrant
Oenanthe oenanthe	H93326	Helgoland	Germany	Vogelwarte Helgoland, Wilhelmshaven, Heiko Schmaljohann	MT431071	blood	Migrant
Oenanthe oenanthe	H93327	Helgoland	Germany	Vogelwarte Helgoland, Wilhelmshaven, Heiko Schmaljohann	MT431072	blood	Migrant
Oenanthe oenanthe	H93328	Helgoland	Germany	Vogelwarte Helgoland, Wilhelmshaven, Heiko Schmaljohann	MT431073	blood	Migrant
Oenanthe oenanthe	H93331	Helgoland	Germany	Vogelwarte Helgoland, Wilhelmshaven, Heiko Schmaljohann	MT431074	blood	Migrant
Oenanthe oenanthe	H93332	Helgoland	Germany	Vogelwarte Helgoland, Wilhelmshaven, Heiko Schmaljohann	MT431075	blood	Migrant
Oenanthe oenanthe	H93333	Helgoland	Germany	Vogelwarte Helgoland, Wilhelmshaven, Heiko Schmaljohann	MT431076	blood	Migrant
Oenanthe oenanthe	H93334	Helgoland	Germany	Vogelwarte Helgoland, Wilhelmshaven, Heiko Schmaljohann	MT431077	blood	Migrant
Oenanthe oenanthe	H93335	Helgoland	Germany	Vogelwarte Helgoland, Wilhelmshaven, Heiko Schmaljohann	MT431078	blood	Migrant
Oenanthe oenanthe	H93336	Helgoland	Germany	Vogelwarte Helgoland, Wilhelmshaven, Heiko Schmaljohann	MT431079	blood	Migrant
Oenanthe oenanthe	H93337	Helgoland	Germany	Vogelwarte Helgoland, Wilhelmshaven, Heiko Schmaljohann	MT431080	blood	Migrant
Oenanthe oenanthe	H93338	Helgoland	Germany	Vogelwarte Helgoland, Wilhelmshaven, Heiko Schmaljohann	MT431081	blood	Migrant
Oenanthe oenanthe	H93339	Helgoland	Germany	Vogelwarte Helgoland, Wilhelmshaven, Heiko Schmaljohann	MT431082	blood	Migrant

Oenanthe oenanthe	H93340	Helgoland	Germany	Vogelwarte Helgoland, Wilhelmshaven, Heiko Schmaljohann	MT431083	blood	Migrant
Oenanthe oenanthe	H93341	Helgoland	Germany	Vogelwarte Helgoland, Wilhelmshaven, Heiko Schmaljohann	MT431084	blood	Migrant
Oenanthe oenanthe	H93342	Helgoland	Germany	Vogelwarte Helgoland, Wilhelmshaven, Heiko Schmaljohann	MT431085	blood	Migrant
Oenanthe oenanthe	H93343	Helgoland	Germany	Vogelwarte Helgoland, Wilhelmshaven, Heiko Schmaljohann	MT431086	blood	Migrant
Oenanthe oenanthe	H93344	Helgoland	Germany	Vogelwarte Helgoland, Wilhelmshaven, Heiko Schmaljohann	MT431087	blood	Migrant
Oenanthe oenanthe	H93345	Helgoland	Germany	Vogelwarte Helgoland, Wilhelmshaven, Heiko Schmaljohann	MT431088	blood	Migrant
Oenanthe oenanthe	H93346	Helgoland	Germany	Vogelwarte Helgoland, Wilhelmshaven, Heiko Schmaljohann	MT431089	blood	Migrant
Oenanthe oenanthe	H93347	Helgoland	Germany	Vogelwarte Helgoland, Wilhelmshaven, Heiko Schmaljohann	MT431090	blood	Migrant
Oenanthe oenanthe	H93348	Helgoland	Germany	Vogelwarte Helgoland, Wilhelmshaven, Heiko Schmaljohann	MT431091	blood	Migrant
Oenanthe oenanthe	H93349	Helgoland	Germany	Vogelwarte Helgoland, Wilhelmshaven, Heiko Schmaljohann	MT431092	blood	Migrant
Oenanthe oenanthe	H93350	Helgoland	Germany	Vogelwarte Helgoland, Wilhelmshaven, Heiko Schmaljohann	MT431093	blood	Migrant
Oenanthe oenanthe	H93351	Helgoland	Germany	Vogelwarte Helgoland, Wilhelmshaven, Heiko Schmaljohann	MT431094	blood	Migrant
Oenanthe oenanthe	H93352	Helgoland	Germany	Vogelwarte Helgoland, Wilhelmshaven, Heiko Schmaljohann	MT431095	blood	Migrant
Oenanthe oenanthe	H93353	Helgoland	Germany	Vogelwarte Helgoland, Wilhelmshaven, Heiko Schmaljohann	MT431096	blood	Migrant
Oenanthe oenanthe	H93355	Helgoland	Germany	Vogelwarte Helgoland, Wilhelmshaven, Heiko Schmaljohann	MT431097	blood	Migrant
Oenanthe oenanthe	H93356	Helgoland	Germany	Vogelwarte Helgoland, Wilhelmshaven, Heiko Schmaljohann	MT431098	blood	Migrant
Oenanthe oenanthe	H93357	Helgoland	Germany	Vogelwarte Helgoland, Wilhelmshaven, Heiko Schmaljohann	MT431099	blood	Migrant
Oenanthe oenanthe	H93358	Helgoland	Germany	Vogelwarte Helgoland, Wilhelmshaven, Heiko Schmaljohann	MT431100	blood	Migrant
Oenanthe oenanthe	G2265	Crete	Greece	Ristow	MT431101	Tissue	Migrant

Oenanthe oenanthe	G2269	Crete	Greece	Ristow	MT431102	Tissue	Migrant
Oenanthe oenanthe	G8605	Kreta	Greece	Ristow	MT431103	Tissue	Migrant
Oenanthe oenanthe	G11501	Kreta	Greece	Ristow	MT431104	Tissue	Migrant
Oenanthe oenanthe	G19615	Crete	Greece	Ristow	MT431105	Tissue	Migrant
Oenanthe oenanthe	G19616	Crete	Greece	Ristow	MT431106	Tissue	Migrant
Oenanthe isabellina	O4325		Israel	Wittmann/Heidrich		Tissue	Unknown

Table S3 Information about the sequences in the study obtained from GenBank

Reference	Species	Sample no.	Location	GenBank accession 16S	GenBank accession COX1
Randler, 2012/Hebert,2004	*O. oenanthe*	1B-3210	Canada		AY666389
Randler, 2012/Kerr,2007	*O. oenanthe*	USNM 627617	Iceland		DQ433051.1
Aliabadian,2007	*O. o. libanotica*	BMNH A/2005.2.9	Iran	DQ683467	DQ683501
Aliabadian,2007	*O. o. libanotica*	BMNH A/2005.2.4	Iran	DQ683468	DQ683502
Aliabadian,2007	*O. o. libanotica*	MIUT2003-81	Iran	DQ683469	DQ683503
Aliabadian,2007	*O. o. libanotica*	BMNH A/2005.2.10	Iran	DQ683470	DQ683504
Aliabadian,2007	*O. seebohmi*	MIUT2003-83	Morocco	DQ683471	DQ683505
van Oosten,2016	*O. oenanthe*	WE13 005	Netherlands	KX352414.1	KX352390.1
van Oosten,2016	*O. oenanthe*	WE13 007	Netherlands	KX352415.1	KX352391.1
van Oosten,2016	*O. oenanthe*	WE13 015	Netherlands	KX352416.1	
van Oosten,2016	*O. oenanthe*	WE13 024	Netherlands	KX352417.1	KX352392.1
van Oosten,2016	*O. oenanthe*	WE13 029	Netherlands	KX352418.1	KX352393.1
van Oosten,2016	*O. oenanthe*	WE13 054	Netherlands	KX352419.1	KX352394.1
van Oosten,2016	*O. oenanthe*	WE13 065	Netherlands	KX352420.1	
van Oosten,2016	*O. oenanthe*	WE13 075	Netherlands	KX352421.1	KX352395.1
van Oosten,2016	*O. oenanthe*	WE13 080	Netherlands	KX352422.1	KX352396.1
van Oosten,2016	*O. oenanthe*	WE13 088	Netherlands	KX352423.1	KX352397.1
van Oosten,2016	*O. oenanthe*	WE13 104	Netherlands	KX352424.1	KX352398.1
van Oosten,2016	*O. oenanthe*	WE13 111	Netherlands	KX352425.1	
van Oosten,2016	*O. oenanthe*	WE13 114	Netherlands	KX352426.1	
van Oosten,2016	*O. oenanthe*	WE13 123	Netherlands	KX352427.1	KX352399.1
van Oosten,2016	*O. oenanthe*	WE13 133	Netherlands	KX352428.1	KX352400.1
van Oosten,2016	*O. oenanthe*	WE13 143	Netherlands	KX352429.1	KX352401.1
van Oosten,2016	*O. oenanthe*	WE13 149	Netherlands	KX352430.1	KX352402.1
van Oosten,2016	*O. oenanthe*	WE13 155	Netherlands	KX352431.1	KX352403.1

van Oosten,2016	*O. oenanthe*	WE13 161	Netherlands	KX352432.1	KX352404.1
van Oosten,2016	*O. oenanthe*	WE13 167	Germany	KX352433.1	KX352405.1
van Oosten,2016	*O. oenanthe*	WE13 172	Germany	KX352434.1	
van Oosten,2016	*O. oenanthe*	WE13 176	Germany	KX352435.1	KX352406.1
van Oosten,2016	*O. oenanthe*	WE13 177	Germany	KX352436.1	KX352407.1
van Oosten,2016	*O. oenanthe*	WE13 181	Germany	KX352437.1	KX352408.1
van Oosten,2016	*O. oenanthe*	WE13 184	Germany	KX352438.1	KX352409.1
van Oosten,2016	*O. oenanthe*	WE13 193	Germany	KX352439.1	KX352410.1
van Oosten,2016	*O. oenanthe*	WE13 201	Germany	KX352440.1	KX352411.1
van Oosten,2016	*O. oenanthe*	WE13 204	Germany	KX352441.1	KX352412.1
van Oosten,2016	*O. oenanthe*	WE13 212	Germany	KX352442.1	KX352413.1
Johnsen,2010	*O. oenanthe*		Sweden_Uppsala		GU571994.1
Johnsen,2010	*O. oenanthe*		Sweden_Orebro		GU571993.1
Johnsen,2010	*O. oenanthe*		Norway_Telemark		GU571512.1
Johnsen,2010	*O. oenanthe*		Norway_Oppland		GU571511.1
Johnsen,2010	*O. oenanthe*		Norway_Rogaland		GU571510.1
Johnsen,2010	*O. oenanthe*		Norway_Vest-Agder		GU571509.1
Kerr, 2009	*O. oenanthe*		Mongolia_Dzavhan		GQ482270.1
Kerr, 2009	*O. oenanthe*		Russia_Rossiya		GQ482266.1
Kerr, 2009	*O. oenanthe*		Russia_Rossiya		GQ482269.1
Kerr, 2009	*O. oenanthe*		Kazakhstan		GQ482268.1
Kerr, 2009	*O. oenanthe*		Russia_Magadanskaya		GQ482267.1
Li, 2016	*O. isabellina*		China_Qinghai	Complete mitochondria genome NC_040290.1	
